# Etching-Chemistry-Driven Ruthenium Doping on Ti_3_C_2_T_*x*_ MXene for Optimizing Electrochemical Performance

**DOI:** 10.1021/acsnanoscienceau.5c00136

**Published:** 2025-10-21

**Authors:** Shanna Marie M. Alonzo, Jared Kinyon, Binod K. Rai, Gayani Pathiraja, Bishnu Prasad Bastakoti

**Affiliations:** † Department of Chemistry, 3616 North Carolina A&T State University,1601 East Market Street. Greensboro, North Carolina 27411, United States; ‡ 1073 Savannah River National Laboratory, Aiken, South Carolina 29808, United States; § Department of Nanoscience, Joint School of Nanoscience and Nanoengineering, 14616 University of North Carolina at Greensboro, 2907 East Gate City Boulevard, Greensboro, North Carolina 27401, United States

**Keywords:** MXene, 2D surface engineering, etching chemistry, ruthenium doping, energy storage, pseudocapacitance

## Abstract

We demonstrate that the etching chemistry used during MXene synthesis from Ti_3_AlC_2_ MAX phase significantly influences surface functionalization and structural vacancies, which in turn affect ruthenium (Ru) ion interactions. Using hydrofluoric acid (HF) and ammonium bifluoride (NH_4_HF_2_) as etchants, we obtained MXene surfaces with distinct functional groups and Ti vacancies that impact Ru ion interactions and electrochemical performance. Both MXene variants (labeled MX­(H) and MX­(N), respectively) exhibited negative zeta potentials in their pristine state, but upon the addition of Ru the zeta potential for MX­(H) reached 12.9 mV while that for MX­(N) remained negative at −6.4 mV. This adsorption resulted in a 14.4-fold increase in the specific capacitance of MX­(H)/Ru compared to pristine MX­(H), whereas MX­(N)/Ru exhibited only a 4.4-fold increase over its pristine counterpart. X-ray diffraction analysis identified the formation of ammonium titanium oxide fluoride, (NH_4_)_3_TiOF_5_, on MX­(N), which likely contributed to its reduced Ru adsorption. X-ray photoelectron spectroscopy suggested the presence of Ti vacancies in both MXene variants; however, their behavior toward Ru accommodation differed markedly, with MX­(H) showing the most obvious shift in the Ti 2p peak in the XPS survey spectrum, while MX­(N) showed the most obvious shift in the C 1s peak. Electron paramagnetic resonance spectroscopy further demonstrated a distinct alteration in the spectral signatures of MX­(H) upon Ru addition, in contrast to the negligible changes in MX­(N), indicating effective passivation of the Ti defect sites in MX­(H) via vacancy-assisted Ru doping. Cyclic voltammetry showed that Ru-incorporated MX­(H) nanocomposites exhibit more efficient redox-active sites, as reflected in their higher capacitance values. These findings highlight the pivotal role of MXene surface chemistry in controlling cation adsorption, providing valuable insights for the rational design of high-performance electrodes.

## Graphical abstract

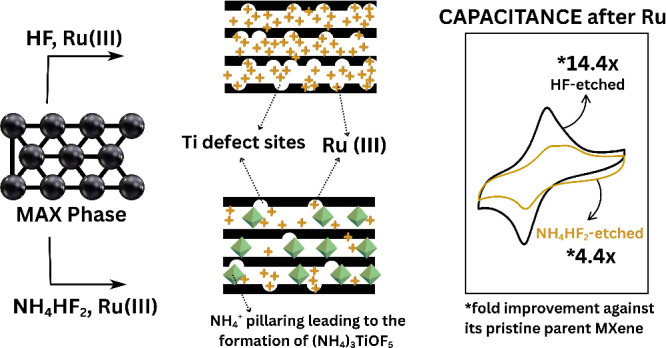

## Introduction

MXenes, which are derived from selective etching of the A element in layered MAX phases (M_*n*+1_AX_*n*_, where M = early transition metal, A = group IIIA or IVA element, and X = C and/or N), have high electrical conductivity, hydrophilicity, and abundant surface terminations (=O, —OH, —F), making them promising for applications in catalysis, sensing, environmental remediations, and electrochemical energy storage.^1,2^ Despite their advantages, pristine MXenes often suffer from suboptimal electrochemical performance due to restacking of their 2D flakes, which drastically impedes ion accessibility and can reduce diffusion rates by up to 8–20 orders of magnitude.^3,4^ Strategies to mitigate this limitation have focused on modulating surface terminations and expanding interlayer spacing, mainly via intercalation or pillaring with various agents.^5–9^ Notably, the incorporation of redox-active species such as transition metals or metal oxides and sulfides has emerged as an innovative approach to boost electrochemical performance via reversible faradaic reactions, particularly in energy storage applications.^9–15^ However, the effectiveness of such modifications is intricately linked to the physicochemical properties of the MXene surface, which in turn are governed by the synthesis route, particularly the etching chemistry.

Recent studies suggest that variations in the etching process, such as using different etching agents, can significantly alter the surface termination profile, chemical properties, and performance of the resulting MXenes.^16–19^ Density functional theory computations have shown that bare Ti_3_C_2_ without surface terminations exhibited a low energy barrier for Li diffusion and a high Li storage capacity when used as an anode for Li-ion batteries, whereas its fluorinated and hydroxylated derivatives hindered Li transport and decreased Li storage capacity.^20^ It has also been demonstrated that the atomic defects in HF-etched MXenes can be tuned by varying the acid concentration, with higher HF concentration leading to more vacancy clusters.^21^ This has been confirmed in another study which additionally showed that MXene with the most abundant Ti vacancies provided strong interactions with noble metals such as Ru, Ir, and Pt.^22^ Collectively, these findings demonstrate that the etching process plays a critical role in determining the properties of MXene; however, the link between etching conditions and the incorporation of adsorbates or dopants remains an open area for further investigation. Unlike typical 2D materials, MXenes possess complex surface terminations that could interact with their intrinsic defects, making it challenging to establish a straightforward link between their composition and functional behavior.^23^ In this study, we explore how etching chemistry influences the incorporation of ruthenium species into Ti_3_C_2_T_*x*_ MXenes through both adsorption and doping pathways by systematically comparing HF-etched and NH_4_HF_2_-etched MXene variants. We elucidate how surface charge, interlayer spacing, functional group composition, and surface defects modulate Ru ion uptake and electrochemical activity. Our results reveal that HF-etched MXenes, with more open structures and abundant Ti vacancies, exhibit better Ru integration and enhanced pseudocapacitive behavior. In contrast, ammonium intercalation in NH_4_HF_2_-etched MXenes and the formation of ammonium titanium oxide fluoride, (NH_4_)_3_TiOF_5_, hinder Ru incorporation due to reduced surface reactivity and blocked adsorption sites. These findings highlight the crucial role of etching chemistry in tailoring MXene surfaces to facilitate redox-active adsorption and doping, providing new design strategies for high-performance, electrochemically active materials for energy storage and related applications.

## Experimental Section

### Materials

MAX phase titanium aluminum carbide/Ti_3_AlC_2_ (Sigma-Aldrich), hydrofluoric acid/HF (Thermo Scientific), ammonium bifluoride/NH_4_HF_2_ (Ricca), ruthenium chloride/RuCl_3_ (Thermo Scientific), ethanol (Fisher Chemicals), 1-methyl-2-pyrrolidinone/NMP (Alfa Aesar), poly­(vinylidene fluoride)/PVDF (Aldrich), microporous carbon cloth (Fuel Cell Earth), and sulfuric acid/H_2_SO_4_ (Fisher Chemical) were purchased and used without further purification.

### Synthesis of MXene

MXene was synthesized using two different etching agents: HF and NH_4_HF_2_, labeled as MX­(H) and MX­(N), respectively. To prepare MX­(H), 1.0 g of Ti_3_AlC_2_ (∼5.1 mmol) was gradually added to 10 mL of 51% HF and stirred for 5 h. For MX­(N), 1.0 g of Ti_3_AlC_2_ (∼5.1 mmol) was gradually added to 40 mL of 2 M NH_4_HF_2_ and stirred at 50 °C for 3 days. The elevated temperature and longer reaction time for NH_4_HF_2_ were necessary to remove Al, as it is a milder etchant that effectively removes Al. The resulting MXene was rinsed several times with deionized water until the pH reached approximately 6–7, followed by vacuum drying at 60 °C for 14 h.

### Synthesis of MXene/Ruthenium Nanocomposites

MX­(H) and MX­(N) were used to prepare nanocomposites with Ru. The amount of MXene was kept constant at 100.0 mg while the Ru content was varied (5.0, 25.0, 50.0, and 100.0 mg of RuCl_3_, equivalent to ∼0.024, ∼0.12, ∼0.24, and ∼0.48 mmol of Ru). MXene was dispersed in 20 mL of ethanol, followed by the addition of RuCl_3_. The mixture was stirred using an orbital shaker for 24 h and then dried in an oven at 50 °C for an additional 24 h. The nanocomposites were labeled MX­(H)/Ru-5, MX­(H)/Ru-25, MX­(H)/Ru-50, MX­(H)/Ru-100, MX­(N)/Ru-5, MX­(N)/Ru-25, MX­(N)/Ru-50, and MX­(N)/Ru-100.

### Preparation of Electrodes

A slurry was prepared by mixing 5.0 mg of the nanocomposite, 1.0 mg of PVDF, and 100 μL of NMP. The mixture was sonicated for 1 h and applied to a microporous carbon cloth (1.5 cm × 1.5 cm) using a small brush. The coated carbon cloth was then dried in an oven at 50 °C for 12 h. To determine the final sample loading, the carbon cloth was weighed before and after coating.

### Characterization

The X-ray diffraction (XRD) patterns of samples were examined using a Rigaku MiniFlex 600 diffractometer with a Cu Kα radiation source and a scintillation counter detector. The morphology and elemental distribution of the samples were studied using a JEOL JSM-IT800 Schottky field emission scanning electron microscope (FESEM). Raman spectra were acquired using a Horiba XploRA Raman confocal microscope. Elemental analysis by X-ray photoelectron spectroscopy (XPS) was performed using an Escalab Xi+ XPS instrument. The morphology and the chemical elemental distribution were examined using a JEOL 2100PLUS transmission electron microscope (TEM) with a scanning TEM and energy-dispersive X-ray spectroscopy (STEM/EDS) capability operated at an accelerating voltage of 200 kV. The zeta potential was measured using a Malvern ZEN3600 Zetasizer. Electron paramagnetic resonance (EPR) spectroscopy was carried out at room temperature using an X-band Bruker ESR5000 spectrometer with 5 mm clear-fused quartz tubes. Microwave power was varied to determine the optimum S/N ratio and prevent signal saturation, which was achieved at 10 and 1 mW for MX­(H)- and MX­(N)-based samples, respectively. Acquisition times were set to 5 min, and the magnetic field swept between 280 and 420 mT, while the modulation field was set to 1 G. The low modulation ensured that the narrow line shapes were not distorted. A Biologic VMP3 instrument was used in a three-electrode setup for electrochemical measurements. Ag/AgCl and platinum wire served as the reference electrode and counter electrode, respectively. A 1 M H_2_SO_4_ solution served as the electrolyte. Before electrochemical tests, the working electrodes were presoaked in the electrolyte for 5 h. Following this, the soaking solution was replaced with fresh electrolyte, and a series of tests, including electrochemical impedance spectroscopy (EIS), galvanostatic charge–discharge (GCD), cyclic voltammetry (CV), and cycling stability assessments, were performed. Specific capacitance was calculated from CV data using eq 1 and from GCD data using eq 2:

1 $$C=\frac{\int_{V_{1}}^{V_{2}}I\;dV}{2sm\Delta V}$$

2 $$C=\frac{I\Delta t}{m\Delta V}$$

where *C* is the specific capacitance (F/g), ∫_*V*_1__^*V*_2_^ *I* d*V* is the integral CV curve area (AV), *s* is the scan rate (mV/s), *m* is the mass of the active material (g), Δ*V* is the potential window, *I* is the discharge current (A), and Δ*t* is the discharge time (s).

## Results and Discussion

### Structural Changes from Etching and Ruthenium-Induced Interlayer Expansion

The morphological transformation of the precursor MAX phase (Ti_3_AlC_2_) from a nanolaminated structure essentially resembling a flat, stacked arrangement (Figure S1a) to a more open, accordion-like structure (Figures 1a,b and S1b–e) indicates the successful synthesis of MXenes (Ti_3_C_2_T_*x*_).^24,25^ During etching with HF or NH_4_HF_2_, the aluminum layer in the MAX phase was selectively removed, resulting in a multilayered material with =O, —OH, and —F surface terminations (represented as T_*x*_ in the general structure of MXene).^26,27^ The removal of aluminum was verified by X-ray diffraction (XRD) where the prominent (104) peak of the MAX phase at 2θ = 38.8° disappeared in both MXenes etched with HF and NH_4_HF_2_ (denoted as MX­(H) and MX­(N), respectively), as shown in Figure 1c.^25,28^ When Al was etched out, the periodicity associated with the ordered presence of Al layers within the hexagonal lattice of the parent MAX phase was disrupted, leading to the disappearance of the (104) peak.^25,29,30^ The resulting MXenes showed the characteristic diffraction peaks corresponding to the (002), (004), (006), (008), and (110) planes.^25,31,32^ For MX­(H), these peaks appeared at 2θ of 9.2°, 18.4°, 27.5°, 35.2°, and 61.0°, respectively, while for MX­(N), they were located at 6.8°, 14.0°, 24.0°, 34.7°, and 61.1°. The weakening, broadening, and shifting of peaks relative to the MAX phase, particularly in the (002) and (004) reflections, are indicative of the formation of multilayered MXene.^25,31,33^ Comparing MX­(H) and MX­(N), the diffraction peaks of MX­(H) appear broader; for an instance, its (002) peak has fwhm of 0.99° compared to 0.58° in MX­(N). This overall more pronounced peak broadening could suggest a greater concentration of lattice defects in MX­(H),^34^ which is consistent with the use of a much stronger etchant and is further corroborated by the subsequent XPS and EPR analyses. Furthermore, in MX­(N), the interaction of NH_4_^+^ with the MXene surface could account for the greater shift toward lower 2θ angles, which directly correlates with the interlayer spacing of the nanosheets.^27,35^ The presence of NH_4_^+^ induces a pillaring effect, further expanding the interlayer distance.^27,36,37^ This interaction is supported by the intense peaks observed at 17.4°, 19.9°, and 28.3° in MX­(N), which suggest the formation of ammonium titanium oxide fluoride, (NH_4_)_3_TiOF_5_, in accordance with PDF 00-054-1072. These peaks align with the characteristic (111), (200), and (220) planes of (NH_4_)_3_TiOF_5_. Additional peaks, such as (222) and (400), also appear, with shifts to higher angles upon interaction with MXene. Its presence would subsequently influence the adsorption and doping behavior of ruthenium ions on MX­(N), as demonstrated in the following sections.

**Figure 1:**
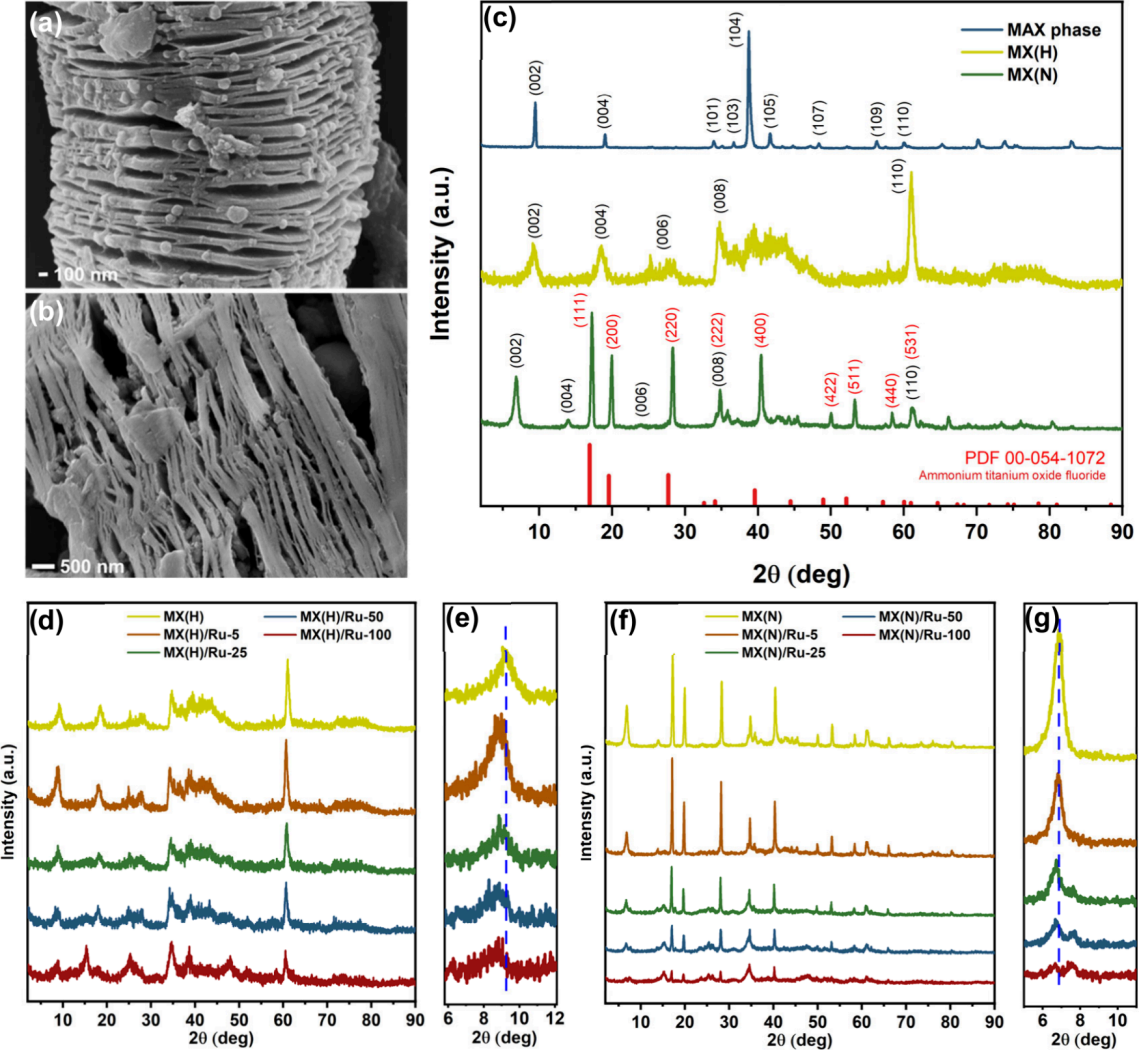
(a, b) SEM images of MXene/Ru composites: (a) MX­(H)/Ru-5 and (b) MX­(N)/Ru-5 (where H and N refer to etching with HF and NH_4_HF_2_, respectively). (c) XRD pattern comparing the parent MAX phase with MX­(H) and MX­(N), plotted with normalized peak intensities, and XRD pattern of (NH_4_)_3_TiOF_5_ according to PDF 00-054-1072. For MX­(N), peaks related to (NH_4_)_3_TiOF_5_ are in red. (d, e) XRD patterns of MX­(H)-based nanocomposites with Ru and the corresponding emphasis on the shifting of (002) peak; (f, g) XRD patterns of MX­(N)-based composites with Ru and the corresponding emphasis on the shifting of (002) peak.

The diffraction pattern of the MXene/ruthenium nanocomposites based on MX­(H) and MX­(N) in different ratios are presented in Figure 1d–g. The XRD patterns of MXene remained largely consistent in all composites, with only a slight leftward shift of the (002) peak (emphasized in Figure 1e,g), showing the influence of the intercalation of Ru ions between MXene sheets. The *c* lattice parameter (*c*-LP), roughly representing the distance between parallel adjacent sheets, was calculated using Bragg’s law (*n*λ = 2*d* sin θ; *c*-LP = 2*d*),^30^ and the values are given in Table 1. MX­(H)-based composites showed *c*-LP from 19.1–20.2 Å, while those of MX­(N)-based composites ranged from 25.8–26.6 Å. However, it is good to note that the splitting of the (002) peak observed in MX­(N) with higher Ru loadings could be due to irregular interlayer spacing throughout the MXene sample.^38^

**Table 1:** Interlayer Distances of MX­(H)- and MX­(N)-Based Nanocomposites with Varying Ru Content

sample	*c* lattice parameter (Å)
MAX phase	19.0343
MX(H)	19.1144
MX(H)/Ru-5	19.9723
MX(H)/Ru-25	19.9364
MX(H)/Ru-50	20.0833
MX(H)/Ru-100	20.1933
MX(N)	25.7838
MX(N)/Ru-5	25.9579
MX(N)/Ru-25	26.2546
MX(N)/Ru-50	26.3132
MX(N)/Ru-100	26.6182

The uniform dispersion of Ru species on the MXene nanosheets was confirmed by transmission electron microscopy (TEM) for both MX­(H)- and MX­(N)-based nanocomposites, as shown in Figures 2 and S2, respectively. For MX­(H), the TEM and HR-TEM images (Figure 2a-b) reveal the formation of well-defined, multilayered stacked nanosheets. Angular-dark field-scanning transmission electron microscopy (ADF-STEM) imaging combined with energy dispersive X-ray spectroscopy (EDS) elemental mapping confirms the uniform distribution of Ti, C, O, and F across the MX­(H) nanosheets (Figure 2c–h). Upon Ru incorporation, the layered architecture remains intact, with observable aggregation of thicker, denser flake assemblies (Figure 2i,j). This is most likely due to positively charged Ru ions attracting neighboring MXene nanosheets, thereby promoting numerous face-to-face interactions that show as denser flake assemblies.^39^ The ADF-STEM image and corresponding elemental maps (Figure 2k–q) clearly demonstrate the homogeneous dispersion of Ru throughout the MXene matrix, indicating effective integration without phase segregation. The corresponding EDS spectra for MX­(H) and MX­(H)/Ru-50 are provided in Figure S3. Almost similar findings were observed for MX­(N) and MX­(N)/Ru-50.

**Figure 2:**
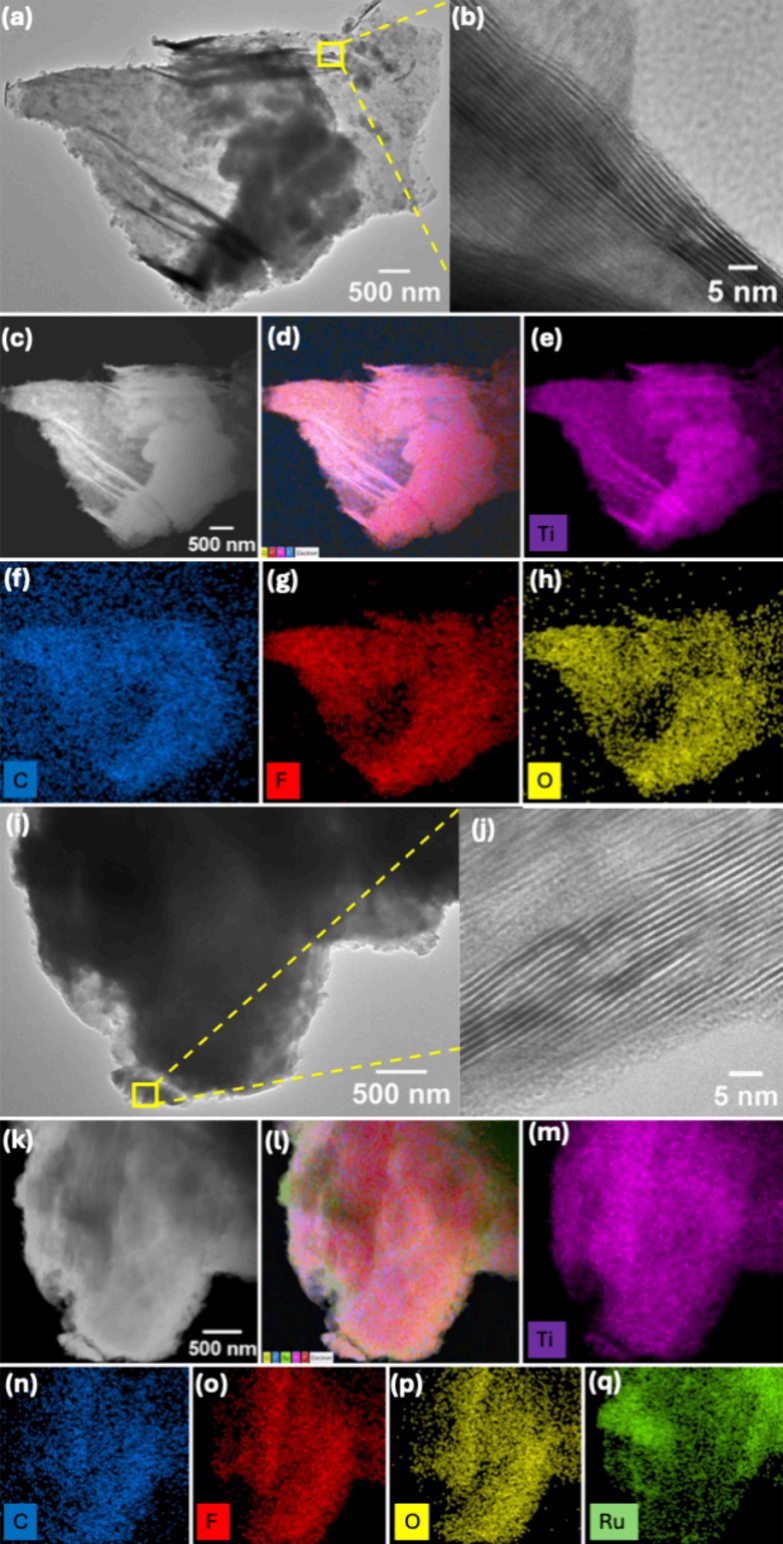
(a, b) TEM image (low magnification) and HR-TEM image taken at 100k× of MX­(H), showing the multilayered structure. (c) ADF-STEM image of MX­(H), (d) corresponding EDS overlay elemental map, and (e–h) individual elemental mappings of Ti, C, F, and O, respectively. (i, j) TEM image (low magnification) and HR-TEM image taken at 200k× of MX­(H)/Ru-50, showing the multilayered structure. (k) ADF-STEM image of MX­(H)/Ru-50, (l) corresponding EDS overlay elemental map, and (m–q) individual elemental mappings of Ti, C, F, O, and Ru, respectively.

### Electronic Modulation and Doping Potential of Ru in MXenes

The Raman spectrum of MXene can be identified into three main regions, as shown in Figure 3a,b. The presence of peaks in these areas, corresponding to the basic Raman modes of the original MAX phase, suggests that the hexagonal crystal structure of the parent MAX phase was preserved in the MXene variants and their nanocomposites.^6,40^ The low-frequency region highlighted in yellow represents group vibrations of Ti, C, and surface groups.^41,42^ This comprises both the in-plane E_g_ and out-of-plane A_1g_ vibration modes of Ti, C, and O. Since this region collectively represents vibrations of the flake as group vibrations, it can be referred to as the flake region. The next region between 200–500 cm^–1^, highlighted in red, represents the E_g_ vibrations of surface groups attached to the Ti atoms. This can be referred to as the T_*x*_ region, in relation to the T_*x*_ term for the surface groups in MXene that result after etching. The final region from about 500–800 cm^–1^ (highlighted in blue) can be assigned to E_g_ and A_1g_ carbon vibrations. Figure 3c shows a graphical illustration of MXene’s Raman modes in the three regions. The direct comparison of the Raman spectra of MX­(H) and MX­(N) in Figure S4 shows distinct profiles in the T_*x*_ region, confirming the difference in their termination groups. Moreover, the ratio of A_1g_(flake) near 200 cm^–1^ to A_1g_(C) at about 600 cm^–1^ is higher in MX­(N), suggesting a lower density of defects.^41^ Comparing the Raman spectra of the MXene/ruthenium nanocomposites with their respective pristine MXene, peak shifting and broadening can be observed. The distinct change in the peak profile within the flake region suggests significant impact in the collective lattice vibrations of the MXene flakes due to the addition of Ru ions.^41,43^ This change, which appears to be either a loss of the peak at approximately 150 cm^–1^ or its merging with the peak at approximately 60 cm^–1^, was more pronounced in MX­(H)-based samples and requires further investigation through in-depth Raman studies to be fully understood. The peak shifting was most prominent in the blue-highlighted region, especially again for the MX­(H)-based nanocomposites which significantly shifted to lower frequencies. Overall, these Raman spectral changes upon the addition of Ru ions confirm their diffusion into the layers of MXene, effectively altering its vibrational behavior.^41,43^ The relatively small ionic radius of the Ru ion (0.68 Å) facilitates its penetration into the interlayer spaces of MXene, which was measured at 19.11 Å for MX­(H) and 25.78 Å for MX­(N) as previously shown in the XRD result.^43,44^ X-ray photoelectron spectroscopy further elucidated the effect of Ru incorporation on the electronic environment of MXene. The survey spectra in Figure 3d-e confirm the elemental composition of the samples and reveal an interesting difference in how MX­(H) and MX­(N) interact with Ru ions. Both pristine MX­(H) and MX­(N) exhibited Ti, C, O, and F peaks, with MX­(N) additionally containing N from ammonium intercalation. Following Ru addition, Ru 3p and Ru 3d peaks emerged at approximately 484.8 and 268.4 eV for MX­(H)/Ru-50, and at 485.9 and 269.7 eV for MX­(N)/Ru-50. Interestingly, the most notable peak shift upon Ru incorporation differed between the two systems: in MX­(H), the Ti 2p peak exhibited the most significant shift, whereas in MX­(N), the shift was observed in the C 1s region. A closer look at the high-resolution C 1s peaks of pristine MX­(H) and MX­(N) in Figure 3f,g, respectively, reveals the possibility of the formation of vacancies during the etching process. The relative intensities of the C—C and C—Ti peaks give qualitative information on Ti vacancy population, i.e., a C—Ti peak with a higher intensity than a C—C peak indicates a nearly vacancy-free structure.^22^ Both MXene variants showed a higher C—C peak, indicating Ti vacancies in both. However, the ratio of C—C to C—Ti peaks is higher in MX­(H), which could mean more clustered vacancy sites.^22^ This opens up to the possibility of vacancy-assisted doping of Ru into the MXene lattice with more available sites in MX­(H)-based nanocomposites. This is further supported by the deconvoluted C 1s spectra of the Ru-containing nanocomposites (MX­(H)/Ru-50 and MX­(N)/Ru-50, shown in Figure S5. Notably, the C—Ti to C—C peak ratio reverses in MX­(H)/Ru-50, with the C—Ti signal now surpassing the C—C peak, pointing to significant restructuring of the electronic environment upon Ru integration (a reversal not observed in MX­(N)/Ru-50). The multivalent nature of Ti in MXene (Figure S6), as already shown extensively in literature, ensures the possibility of charge transfer or redox reactions with metal cations like Ru.^39^ The deconvoluted Ru 3p_1/2_ peaks (Figure S7) show the presence of richer high-valence species Ru^δ+^ in MX­(H)/Ru-50 compared to MX­(N)/Ru-50.^22,45^ The reduction of Ru^δ+^ to a lower valence in the former could have resulted from its interaction with Ti. But even in the absence of a complete electron transfer, the shift of the Ti 2p peak to higher binding energy, as shown in the survey spectrum of MX­(H)/Ru-50, implies a decrease in electron density around Ti atoms. This suggests that the more electronegative Ru draws the electron cloud away from the neighboring Ti sites upon doping. Given the similar d-orbital configurations of Ti and Ru, orbital hybridization is possible, which enhances charge delocalization and facilitates more efficient charge transfer. This advantage becomes evident in the subsequent electrochemical performance.

**Figure 3:**
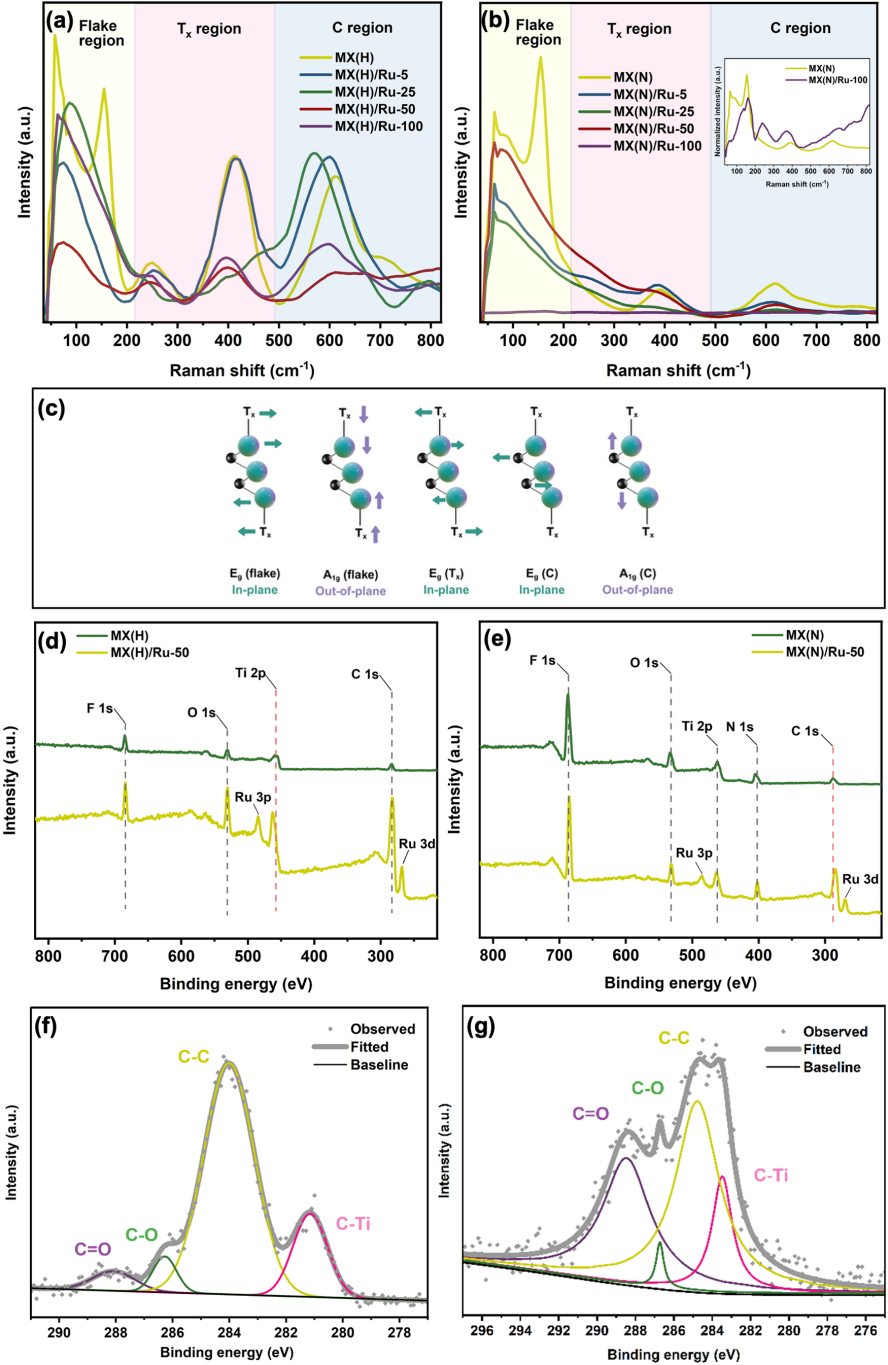
(a, b) Raman spectra of (a) MX­(H)/Ru nanocomposites and (b) MX­(N)/Ru nanocomposites (yellow-highlighted region = flake region; red = T_*x*_ region; blue = C region). The inset in (b) shows normalized spectra of MX­(N) and MX­(N)/Ru-100 for better visualization of peaks for the latter. (c) Graphical illustration of the Raman active modes in MXene. XPS survey spectra of (d) MX­(H) and MX­(H)/Ru-50, showing a shift in the Ti 2p peak toward higher binding energy upon Ru addition, and (e) MX­(N) and MX­(N)/Ru-50, showing a shift in the C 1s peak toward lower binding energy upon Ru addition. (f, g) Deconvoluted C 1s peaks of pristine MX­(H) and MX­(N), respectively.

### EPR-Detected Alteration of Ti Cluster Defects in HF-Etched MXene upon Ruthenium Integration

To further investigate the Ti cluster defects and the associated differing interactions of MX­(H) and MX­(N) with Ru ions previously suggested by XPS results, electron paramagnetic resonance (EPR) spectroscopy was carried out. The EPR spectra (Figure 4) confirmed that Ru incorporation significantly altered the Ti defect sites in MX­(H), whereas MX­(N) showed negligible spectral changes. For MX­(H), the original single Lorentzian peak at 354.6 mT could be fit with a *g* factor of 1.945 and a peak-to-peak width (Δ*H*_pp_) of 2.7 mT, clearly indicating Ti cluster defects. The *g* factor was well within the expected range for Ti defects in Ti_3_C_2_T_*x*_,^22,46,47^ Ti_3–*x*_C_2_T_*y*_,^48^ and Ti_2_CT_*x*_ ^49^ MXenes. The EPR spectrum for MX­(H)/Ru-100 appeared significantly different from its parent MX­(H), showing a broad central peak around 335 mT, a narrow peak superimposed on the broad peak also at 335 mT, and a narrow dip around 354 mT (Figure 4a). Using Easy Spin,^50^ the simulations required a three-spin system with no g-anisotropy to accurately reproduce the experimental EPR spectrum (discussed further in Figure S8). The observed dip was considered to be an independent spin center, justifiably so since its position was very close to the peak found in its parent MX­(H), as emphasized in Figure S9. This suggests that the peak arises from residual Ti defects, i.e., a few sites that remained unoccupied after Ru addition. Additionally, the *g* factor slightly shifted from 1.945 to 1.940, and Δ*H*_pp_ broadened from 2.7 to about 6.3 mT after Ru doping, confirming the alteration of the local electronic environment around the original Ti cluster defects and thus corroborating with Raman and XPS results. Notably, the relative decrease in intensity for the Ti defect peak in EPR parallels with the reversal in the C—Ti to C—C peak ratio in the XPS C 1s spectra, collectively supporting Ru-induced passivation of Ti vacancy sites in MX­(H)-based samples.^22^

**Figure 4:**
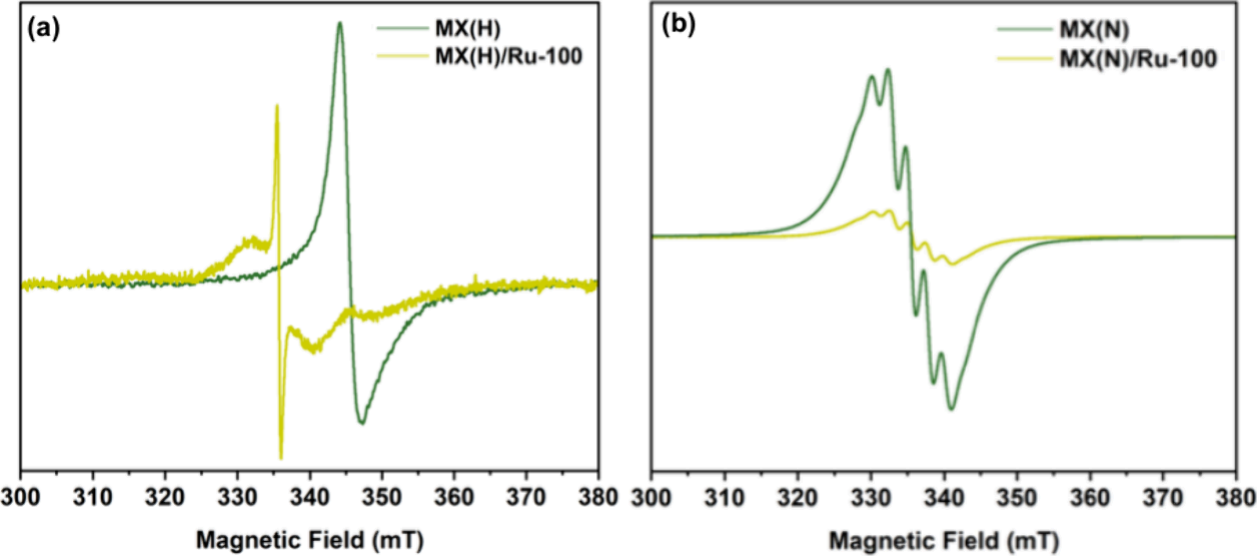
Direct comparisons of the EPR spectra of (a) MX­(H) and MX­(H)/Ru-100 and (b) MX­(N) and MX­(N)/Ru-100

MX­(N) produced a distinctly different EPR spectrum from MX­(H), as shown in Figure 4b. Instead of a single Lorentzian line, seven narrow, evenly spaced lines (*A*_iso_ ≈ 2.3 mT) characteristic of hyperfine splitting were observed, although broadening could be hiding low-intensity peaks in the multiplet. In short, these signals may originate from numerous possible causes within this complex system, although their origin can be narrowed down through simulations and chemical intuition. For example, both the possibility of residual ^27^Al (*I* = 5/2) from the precursor MAX phase and the observed formation of (NH_4_)_3_TiOF_5_ were separately considered as sources of the hyperfine splitting. Regardless, simulations did not support the idea of leftover aluminum or Ti defects interacting locally with ^19^F (*I* = 1/2) (discussed more in Figures S10 and S11). The best spectral fit was achieved when a defect near the TiOF_5_^3–^ octahedron is considered to interact with nitrogen (*I* = 1 for ^14^N and 1/2 for ^15^N) from surrounding ammonium groups. Such an interaction produces a spectrum that looks similar to what was previously reported in the literature for three equivalent ^11^B nuclei (*I* = 3/2) for an N defect site in boron nitride.^51^ Symmetry-wise, it seemed reasonable that a defect in the TiOF_5_^3–^ anion could interact with four equivalent N atoms. Interestingly, the spectrum also fits with three equivalent N atoms, a scenario that would hypothetically produce seven distinct peaks from ^14^N, which is conveniently consistent with the original EPR spectrum. Both simulations, involving three or four N atoms, are presented in Figure S12. As shown in Table S2, *A*_iso_ is nearly equivalent for all nitrogen atoms, which is to be expected if they experience similar ligand environments due to the defect. However, directly comparing both simulations with the experimental data, as shown in Figure S13, modeling with four equivalent N produces a superior fit, especially when considering the overlap with the 340 mT peak. This matches the expected symmetry of ammonium ions near a titanium center for (NH_4_)_3_TiOF_5_. Finally, the negligible difference between the EPR spectra of MX­(N) and MX­(N)/Ru-100 confirms that the addition of Ru had a minimal impact on the Ti defect environment in the original MX­(N), reinforcing the conclusions drawn from XPS analysis. Although Ti defects were also observed, the formation of (NH_4_)_3_TiOF_5_ appears to have impeded effective Ru doping via interaction with Ti vacancies.

### Electrochemical Performance

Working electrodes were prepared from MX­(H)- and MX­(N)-based nanocomposites to evaluate their electrochemical behavior. Cyclic voltammetry (CV) was conducted over a range of potential windows to identify optimal operating limits. Figure 5a,b displays the CV curves from 0.05–0.55 V to 0.05–1.15 V for MX­(H)/Ru-100 and MX­(N)/Ru-100, respectively, while those for the other samples are provided in Figures S14 and S15. At more expansive potential windows, the curves exhibit instability and signs of oxygen evolution, resulting from water electrolysis, as indicated by the abrupt rise in the current response at higher potentials.^52^ Consequently, 0.05–0.75 V was selected as the working range for subsequent tests to avoid unwanted side reactions. The CV profile at this window (insets of Figure 5a,b) deviates from a typical quasi-rectangular shape, indicating energy storage behavior resulting from both electric double layer formation at the electrode surface and pseudocapacitive processes.^53^ The prominent anodic peak at ∼0.40 V and cathodic peak at ∼0.27 V for both MX­(H)/Ru-100 and MX­(N)/Ru-100 confirm the occurrence of redox reactions. These redox peaks are not observed in MX­(H) and MX­(N) but appear in all Ru-containing nanocomposites, including those with the lowest Ru content, i.e., MX­(H)/Ru-5 and MX­(N)/Ru-5 (insets of Figures S14a–d and S15a–d). From this, it can be deduced that the redox reactions primarily arise from the presence of Ru in the nanocomposites. Figure 5c,d shows the CV curves at increasing scan rates for MX­(H)/Ru-50 and MX­(N)/Ru-50, respectively. The corresponding CV curves at increasing scan rates for the other samples are shown in Figures S16 and S17. Using eq 1, the specific capacitance values for MX­(H)/Ru-50 were calculated to be 20.27, 13.19, 11.71, 10.94, 10.44, 10.03, and 9.71 F/g at 2, 20, 40, 60, 80, 100, and 120 mV/s, respectively. For MX­(N)/Ru-50, these values were 10.53, 6.21, 5.34, 4.87, 4.55, 4.35, and 4.23 F/g at the same scan rates. By examining the correlation between specific capacitance (*C*) and scan rate (*s*), the charge storage mechanisms in these electrode materials were distinguished. At lower scan rates, the total capacitance comprises both surface capacitive contributions (electric double-layer capacitance, EDLC) and diffusion-controlled pseudocapacitance, which involves redox reactions, ion intercalation, and adsorption processes. In this approach, the y-intercept of the linear fit from the plot of *s* ^1/2^ versus 1/*C* represents the total capacitance. As the scan rate increases, the contribution of relatively slower pseudocapacitive processes decreases due to ion diffusion limitations, resulting in EDLC becoming the dominant capacitance component.^54,55^ Therefore, the plot of *s* ^–1/2^ versus *C* provides an estimate of the EDLC contribution. This analysis determined that the charge storage consisted of 43.6% capacitive (EDLC) and 56.4% pseudocapacitive (PS) contributions for MX­(H)/Ru-50, while 32.1% EDLC and 67.9% PS contributions for MX­(N)/Ru-50, as shown in Figure S18. These values were not far off from their pristine counterparts, with MX­(H) showing 37.6% EDLC and 62.4% PS, and MX­(N) with 34.7% EDLC, 65.3% PS.

**Figure 5:**
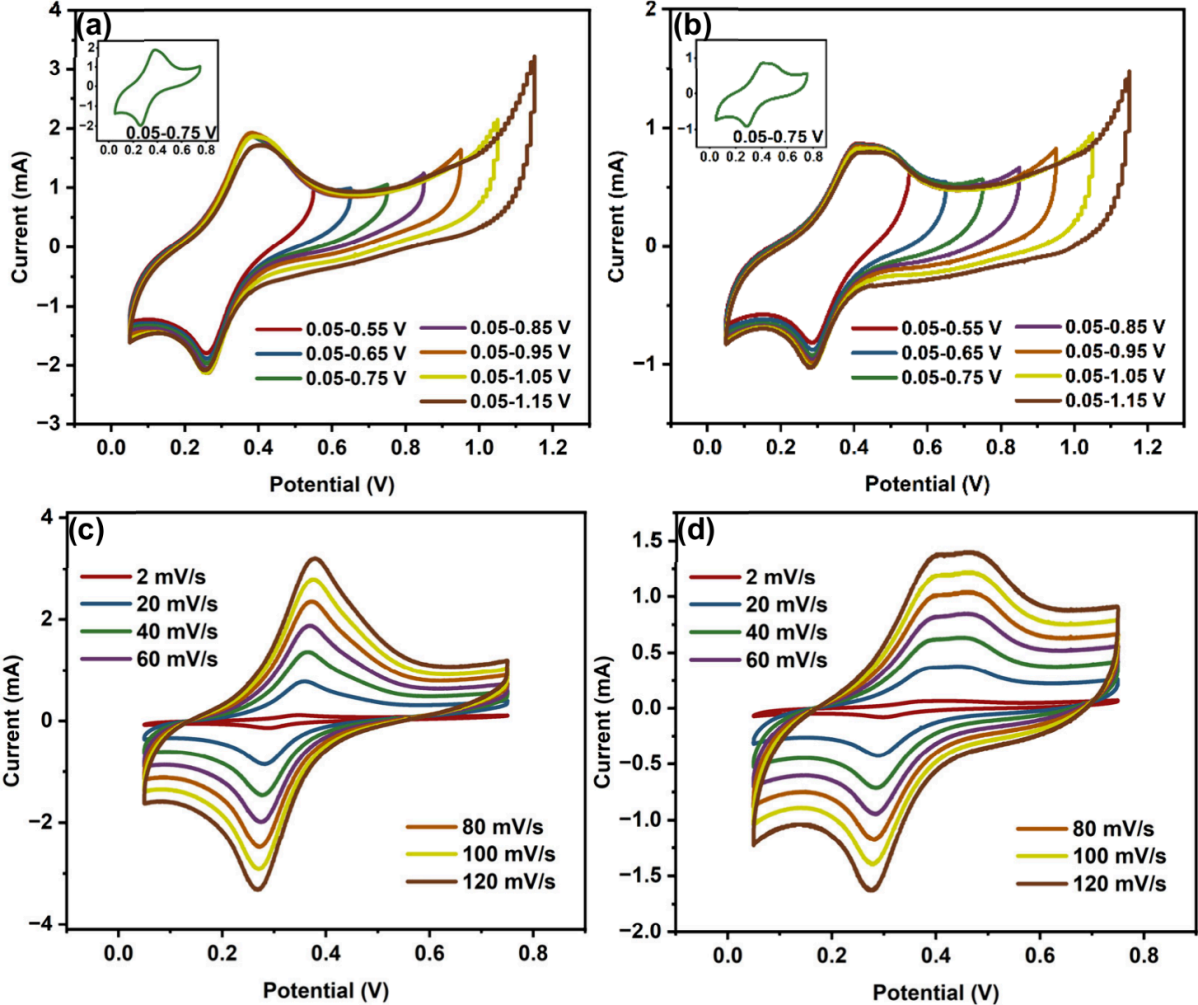
CV curves at different potential windows of (a) MX­(H)/Ru-100 and (b) MX­(N)/Ru-100 and CV curves at increasing scan rates of (c) MX­(H)/Ru-50 and (d) MX­(N)/Ru-50.

A comparative CV graph comparing MX­(H)/Ru-50 and MX­(N)/Ru-50 is presented in Figure 6a, highlighting the difference in the current density of the samples. Notably, MX­(H)/Ru-50 exhibits more pronounced redox peaks, while the broader and shouldered oxidation peak observed in MX­(N)/Ru-50 suggests a relatively sluggish redox kinetics arising from charge-transfer resistance.^56^ The galvanostatic charge/discharge (GCD) curves recorded at a current density of 1 A/g (Figure 6b) further confirm the presence of redox reactions, with the deviation from a symmetric triangular shape indicating pseudocapacitive behavior.^57,58^ The redox peaks in CV are manifested as plateaus in GCD (around 40–55 s in MX­(H)/Ru-50) and indicate deep intercalation and faradaic reactions in the material.^57^ Using eq 2, the specific capacitances from the GCD curves of MX­(H)/Ru-50 and MX­(N)/Ru-50 were found to be 63.6 F/g and 22.8 F/g, respectively. Figure 6c shows the specific capacitance of all MX­(H)- and MX­(N)-based nanocomposites at different scan rates. Introducing Ru into both MXene variants enhanced capacitance, with the extent of improvement depending on Ru content and the MXene synthesis route. MX­(H)/Ru-100 exhibited the highest capacitance of 25.11 F/g at 2 mV/s, while MX­(N)/Ru-100 reached only 8.69 F/g under the same conditions. Notably, the capacitance enhancement was more pronounced for MX­(H)-based nanocomposites, owing to differences in MXene surface chemistry. Both MXene types demonstrated that moderate Ru loading (100:50) provided the best balance between high capacitance and good rate capability. While 100:100 compositions achieved higher capacitance at low scan rates, their performance declined at higher scan rates, suggesting potential ion transport limitations due to Ru aggregation. On the other hand, 100:5 and 100:25 exhibited minimal improvement over pristine MXene, indicating insufficient active sites for redox reactions. Notably, MX­(H)/Ru generally outperformed MX­(N)/Ru, highlighting better electrochemical compatibility of the HF-etched structure with Ru. The difference in capacitance suggests that MX­(H) allows better Ru incorporation, leading to more redox-active sites, whereas MX­(N) may experience reduced Ru integration efficiency. This aligns with the XPS and EPR findings of Ru interacting with Ti defect sites in MX­(H)-based nanocomposites, potentially leading to d-orbital hybridization and more efficient charge transfer. Overall, the 100:50 composition emerged as the most promising Ru loading for both MXene types, striking a balance between enhanced capacitance and good rate performance.

**Figure 6:**
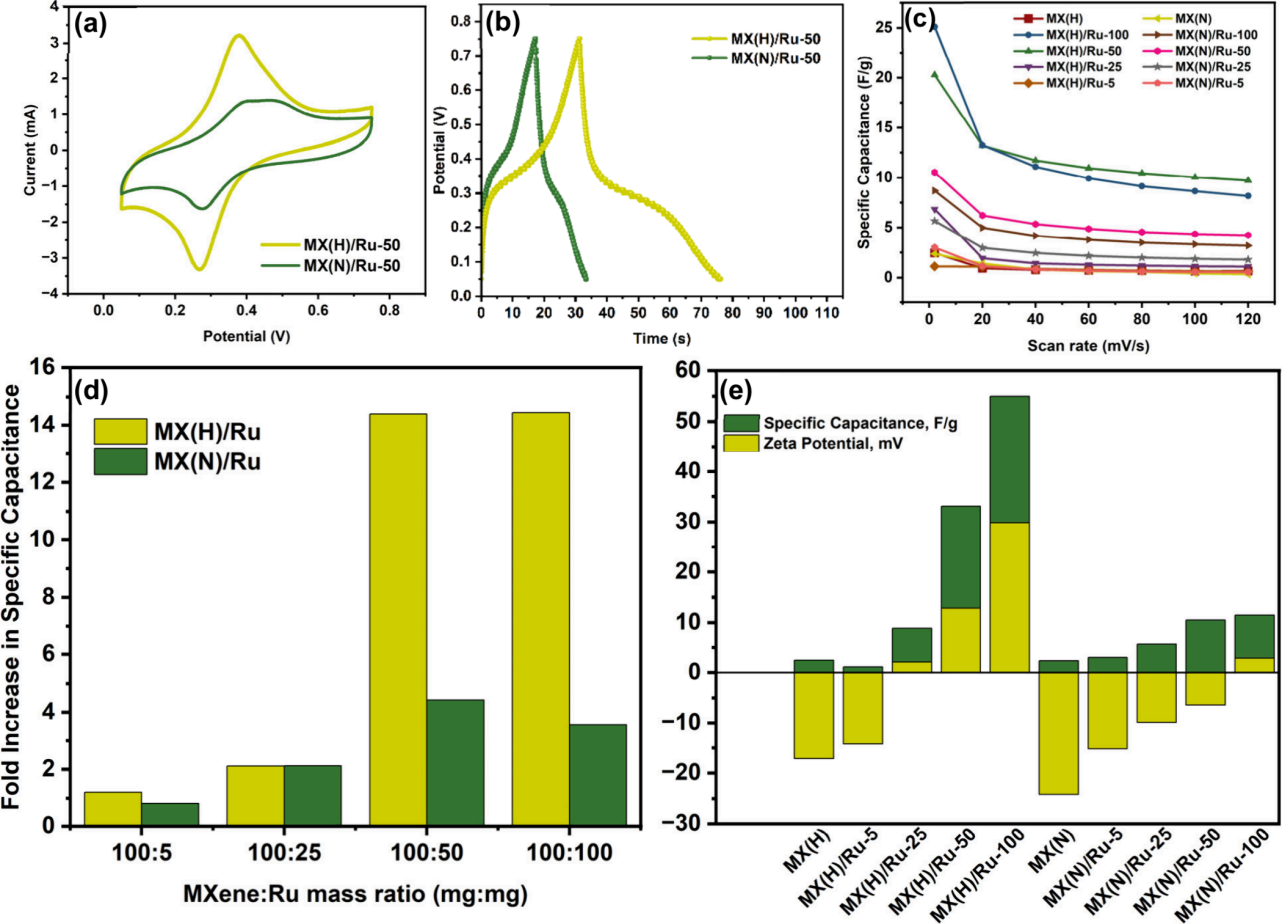
(a) Comparative CV and (b) GCD curves for MX­(H)/Ru-50 and MX­(N)/Ru-50, (c) Specific capacitance as a function of scan rate for all samples, (d) Fold increase in specific capacitance of MX­(H)/Ru and MX­(N)/Ru nanocomposites relative to their corresponding pristine MXene at different MXene:Ru mass ratios, and (e) Comparison of specific capacitance and surface charge for all samples.

### Surface Charge, Electrostatic Interactions, and Effect on Pseudocapacitance

In Figure 6d, the impact of Ru loading on the specific capacitance of MXene-based composites was evaluated by comparing the fold increase in capacitance of MX­(H)/Ru and MX­(N)/Ru nanocomposites relative to their respective pristine MXene. At a low Ru loading (100:5), both MX­(H) and MX­(N) exhibited minimal enhancement in capacitance, with fold increases of 1.21 and 0.82, respectively. This suggests that at this ratio, Ru incorporation was insufficient to contribute to redox activity. As the Ru content increased to a 100:25 ratio, both materials showed comparable capacitance enhancement at about 2-fold increase, indicating that at this intermediate loading, the Ru incorporation had a similar effect on charge storage for both MXene variants. However, at higher Ru loadings, a stark contrast emerged between MX­(H) and MX­(N). At 100:50, MX­(H) exhibited a remarkable 14.39-fold increase in capacitance, whereas MX­(N) showed only a 4.43-fold increase. This trend persisted at the 100:100 ratio, where MX­(H) retained a high enhancement factor (14.44), while MX­(N) demonstrated a lower 3.55-fold increase. These results again indicate that Ru incorporation was significantly more effective on MX­(H) than on MX­(N), particularly at higher Ru loadings. This discrepancy can be attributed to the zeta potential analysis, which revealed that the addition of Ru resulted in a significant increase in zeta potential for MX­(H) but only a modest change for MX­(N), as illustrated in Figure 6e. MX­(H) and MX­(N) contain oxygen and fluorine atoms in their elemental composition, as indicated by the XPS surveys. These are from the surface termination of =O, —OH, and —F groups formed during the etching process, which then account for the negative surface charge of the pristine MXene variants.^25,59^ Due to these functional groups, MXene materials exhibit interactions with metal cations similar to those of clays, primarily occurring through electrostatic attractions and coordination bonding.^39^ The strong Ru adsorption on MX­(H) results from its negatively charged surface due to functional groups, which facilitate electrostatic interactions with Ru cations. In contrast, MX­(N) is already preintercalated by ammonium species, as confirmed by XRD analysis, which limits the available adsorption sites for Ru. These ammonium cations functioned as structural pillars between MX­(N) sheets, evidenced by their relatively higher *c*-LP values compared with MX­(H), and most likely hindered additional cationic adsorption of Ru ions. It is noteworthy that the zeta potential of MX­(N) crossed the zero mark only after the highest loading of Ru (100:100), reaching only 2.83 mV, while MX­(H) already reached a positive zeta potential at the 100:25 ratio (2.09 mV) and reached as high as 29.82 mV at 100:100.

Finally, the cycling stability test, conducted over 8000 cycles at 200 mV/s, reveals differences in capacitance retention across the various MXene samples (Figure 7a). Pristine MX­(H) exhibits an increase in capacitance retention of 132.39%, suggesting activation during cycling,^60^ whereas MX­(N) retains only 37.36%, indicating substantial degradation. The incorporation of Ru enhances stability in both variants, with MX­(H)/Ru-100 and MX­(N)/Ru-100 achieving nearly full retention at 100.05% and 102.96%, respectively. Moderate Ru loadings (Ru-25 and Ru-50) also improve stability, particularly for MX­(N), which sees a substantial increase to 95.98% in MX­(N)/Ru-50. However, lower Ru content (Ru-5) provides limited enhancement, with capacitance retention at 74.08% and 61.20% for MX­(H)/Ru-5 and MX­(N)/Ru-5, respectively. The initial and final capacitance values are shown in Table S3. The CV curves shown in Figure 7b–e illustrate how the electrochemical response evolves with cycling for MX­(H)/Ru-5, MX­(H)/Ru-100, MX­(N)/Ru-5, and MX­(N)/Ru-100, respectively. These CV curves were extracted by 100-interval increments over the course of 8000 cycles. The corresponding cycling curves for the remaining samples are provided in Figure S19. Notably, samples with lower Ru content exhibited more pronounced changes in CV shape, particularly during the first few hundred cycles, indicating performance degradation (Figure 7b,d). In contrast, the CV profiles of samples with higher Ru content remain well-preserved even after 8000 cycles, confirming the structural integrity and reversible charge storage behavior of these materials (Figure 7c,e). These findings highlight the role of Ru incorporation in stabilizing the MXene structure by promoting sustained electrochemical activity via redox reactions, making the material durable for long-term use.

**Figure 7:**
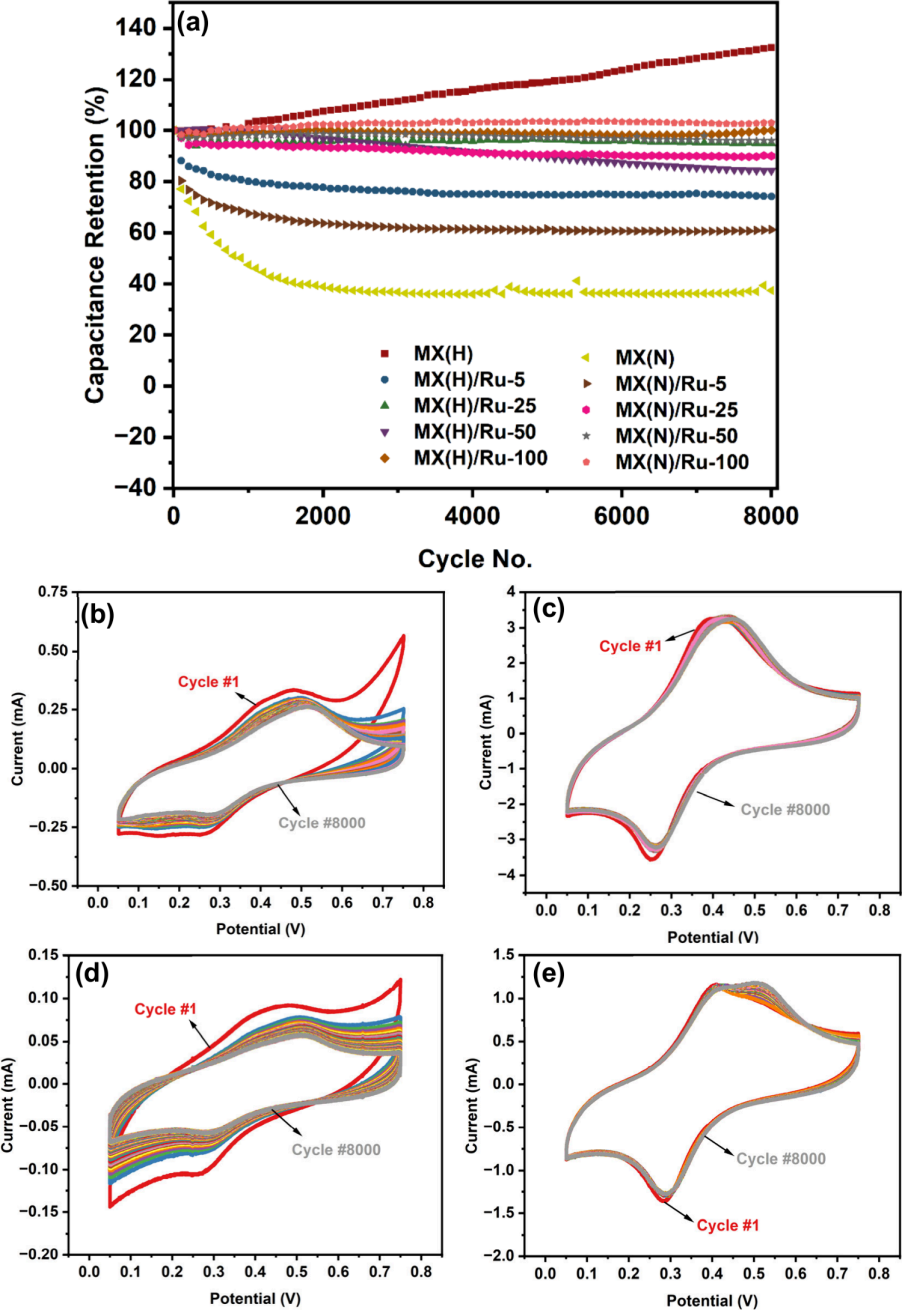
(a) Capacitance retention during cycling stability test for all samples. (b–e) CV curves taken at 100-interval increments over the course of 8000 cycles for (b) MX­(H)/Ru-5, (c) MX­(H)/Ru-100, (d) MX­(N)/Ru-5, and (e) MX­(N)/Ru-100.

## Conclusion

This study gives insight into the nuanced role of synthesis routes and loading of redox-active metal cations on the electrochemical behavior of MXene-based nanocomposites. By contrasting MXene variants synthesized using HF and NH_4_HF_2_, we demonstrate that etching chemistry dictates surface charge, structural accessibility via vacancies, and ion adsorption and doping behavior. Ruthenium incorporation proves more efficient in HF-etched MXene, with surface functional groups and Ti cluster vacancies facilitating stronger electrostatic interactions and improved Ru ion doping, as shown in XPS and EPR analyses. This leads to higher redox activity and enhanced structural stability during long-term cycling. In contrast, ammonium intercalation in NH_4_HF_2_-etched MXene and the formation of ammonium titanium oxide fluoride, (NH_4_)_3_TiOF_5_, limit accessible sites for Ru interaction, thereby diminishing the extent of electrochemical performance enhancement. These findings highlight the importance of etchant selection and surface engineering in optimizing MXene-based energy storage materials. The insights gained offer a platform for rational design of high-performance pseudocapacitive and related systems via targeted electronic and interfacial modulation.

## Supplementary Material



## Data Availability

The data are available upon request.

## Notes

SEM images, TEM images, EDS spectra, Raman spectra, XPS spectra, EPR spectra, and electrochemical measurements (PDF)
