# Supplementary material for: Etching-Chemistry-Driven Ruthenium Doping on Ti3C2T x MXene for Optimizing Electrochemical Performance
Source: ACS Nanosci Au. 2025 Oct 21;5(6):585–97. doi: 10.1021/acsnanoscienceau.5c00136 (PMC12715641; doi:10.1021/acsnanoscienceau.5c00136)
Supplement: Supplementary file 1 [file ng5c00136_si_001.pdf]

Etching Chemistry-Driven Ruthenium Doping on $Ti_3C_2T_x$ MXene for Optimizing Electrochemical Performance

Shanna Marie M. Alonzo[1], Jared Kinyon[2], Binod K. Rai[2], Gayani Pathiraja[3], and Bishnu Prasad Bastakoti[1]*

[1]Department of Chemistry, North Carolina A&T State University,1601 E. Market St. Greensboro, NC 27411, USA

[2]Savannah River National Laboratory, Aiken, SC 29808, USA

[3]Department of Nanoscience, Joint School of Nanoscience and Nanoengineering, University of North Carolina at Greensboro, 2907 East Gate City Blvd, Greensboro, NC 27401, USA

bpbastakoti@ncat.edu

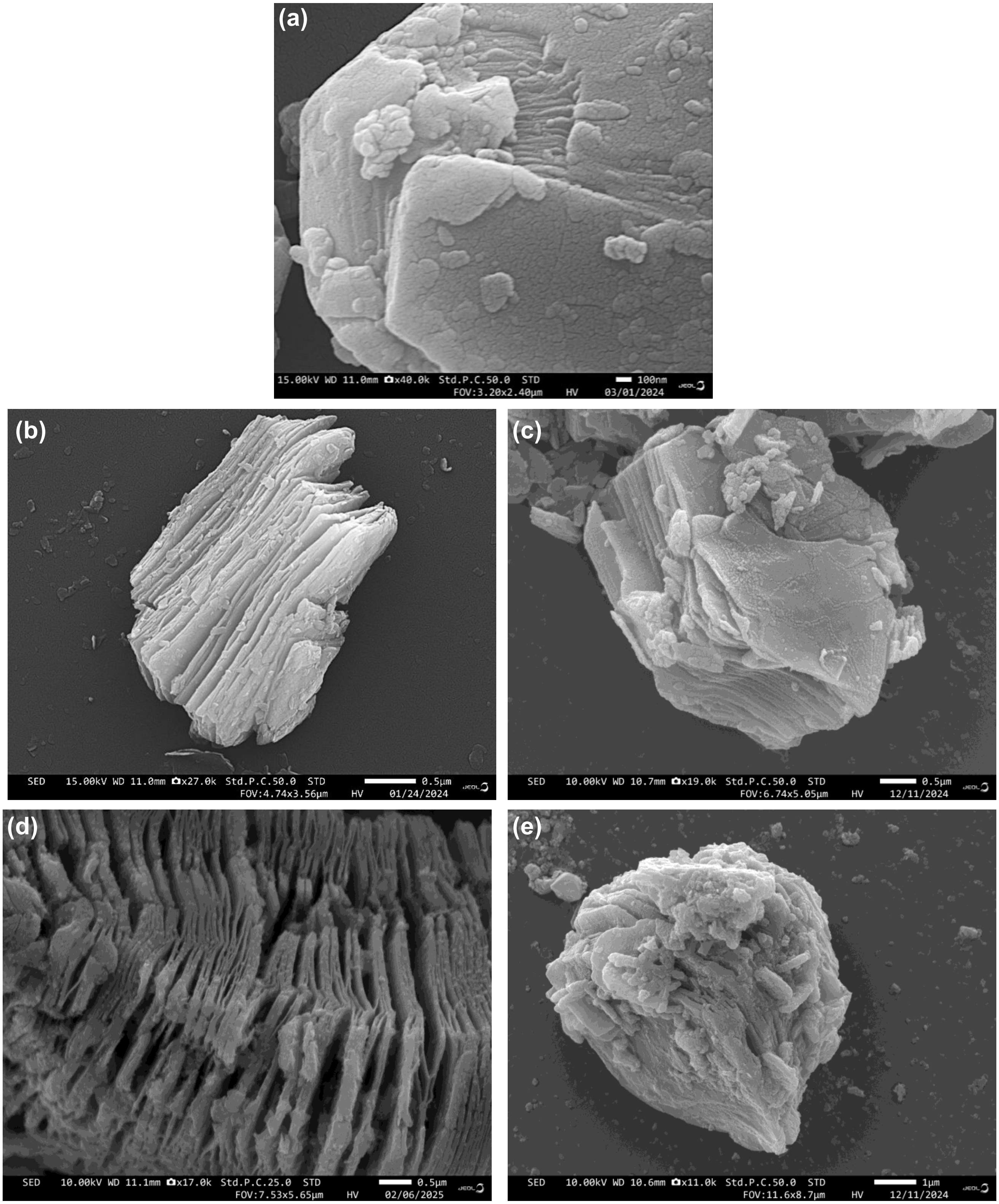

Figure S1. SEM image of the (a) as-received MAX phase Ti_3AlC_2, (b) MX(H), (c) MX(N), (d) MX(H)/Ru-50, and (e) MX(N)/Ru-50

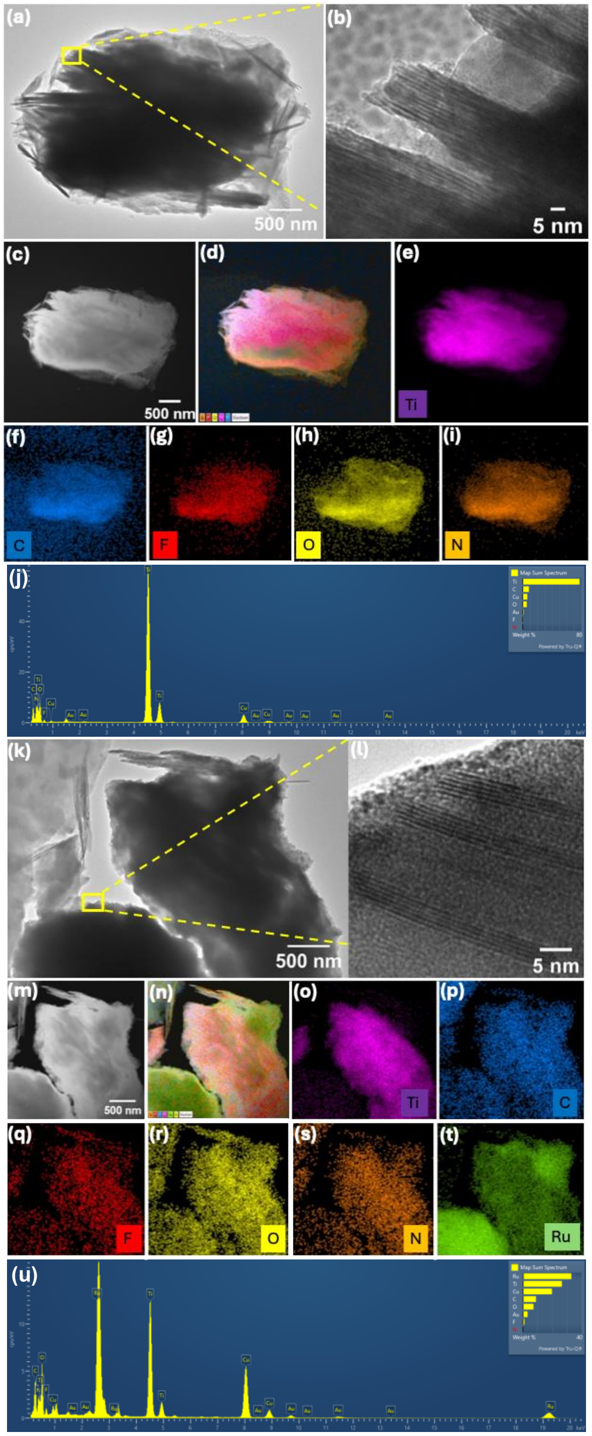

Figure S2. (a-b) TEM image (low magnification) and HR-TEM image taken at 100kx of MX(N), showing the multilayered structure; (c) the ADF-STEM image of MX(N); (d) corresponding EDS overlay elemental map, and (e–i) individual elemental mapping of Ti, C, F, O, and N respectively; (j) corresponding EDS spectra of MX(N); (k-l) TEM image (low magnification) and HR-TEM image taken at 200kx of MX(N)/Ru-50, showing the multilayered structure; (m) the ADF-STEM

image of MX(N)/Ru-50; (n) corresponding EDS overlay elemental map; (o–t) individual elemental mapping of Ti, C, F, O, N, and Ru, respectively; and (u) corresponding EDS spectra of MX(N)/Ru-50. The EDS maps seem to suggest the presence of local Ru-rich clusters, thus yielding a high local Ru wt% value. This is in contrast with the more uniformly dispersed Ru on MXene surface found in MX(H).

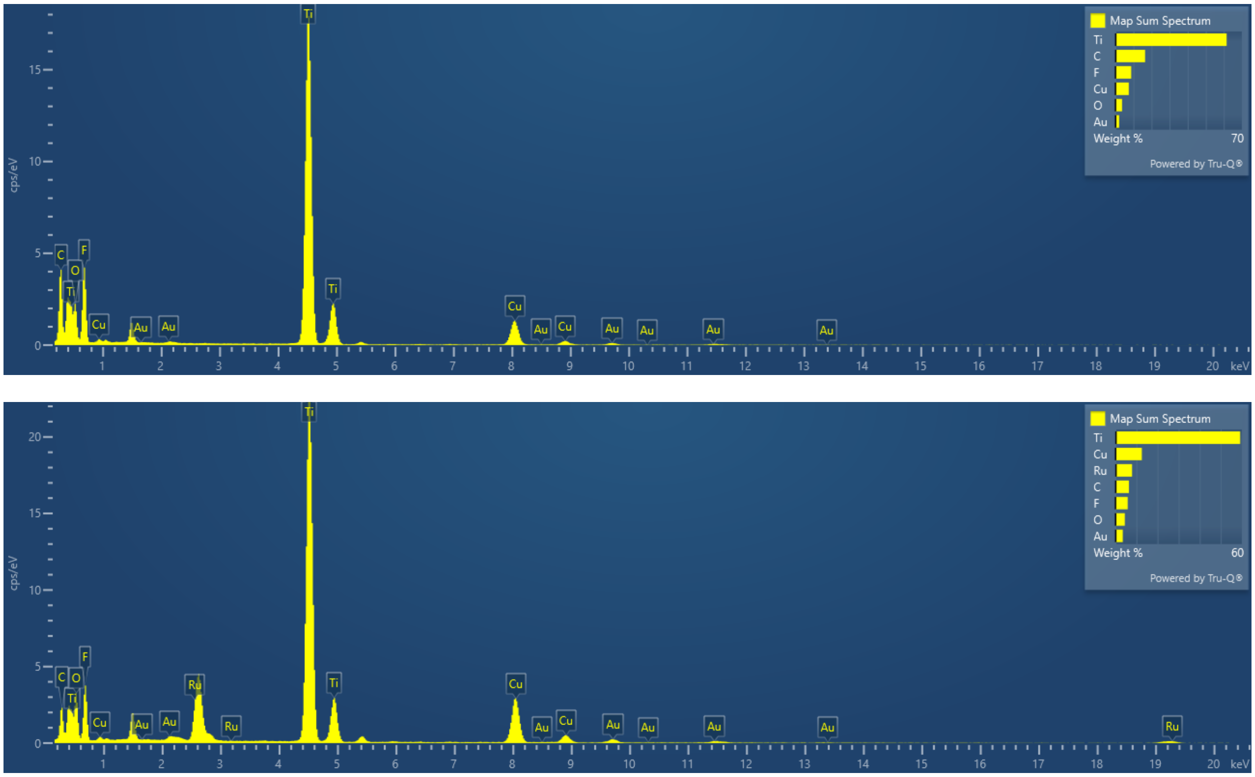

Figure S3. EDS spectra of (a) MX(H) and (b) MX(H)/Ru-50. The Cu signal originates from the TEM grid, Au from the sample holder, and part of the detected C also arises from the grid.

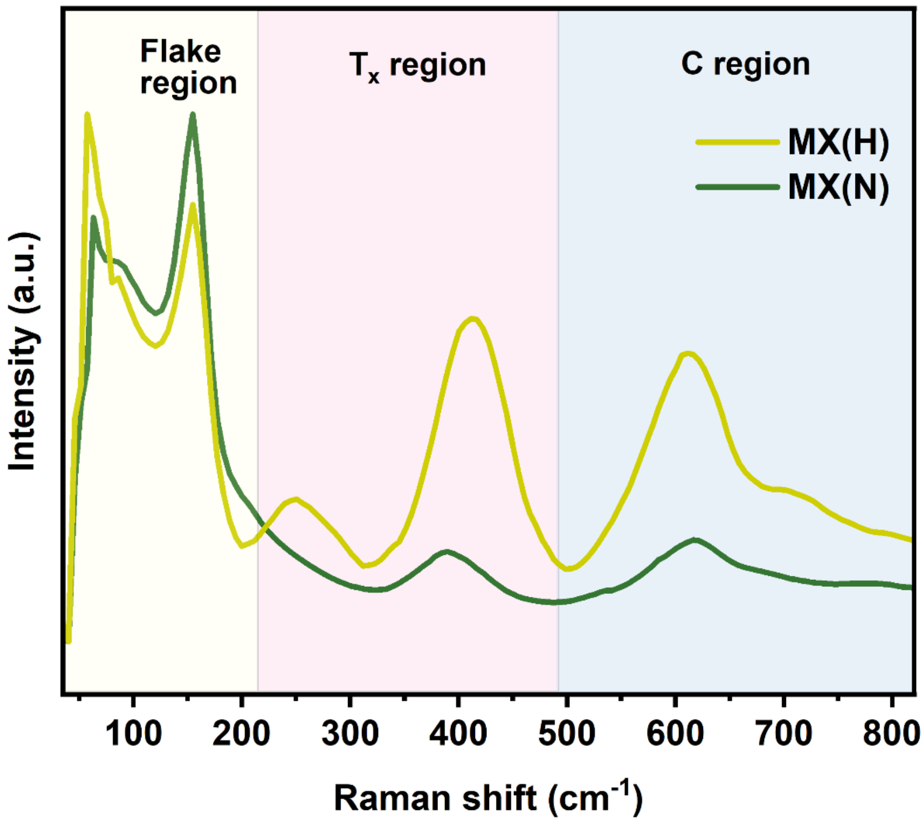

Figure S4. Raman spectra of MX(H) and MX(N) with normalized intensity

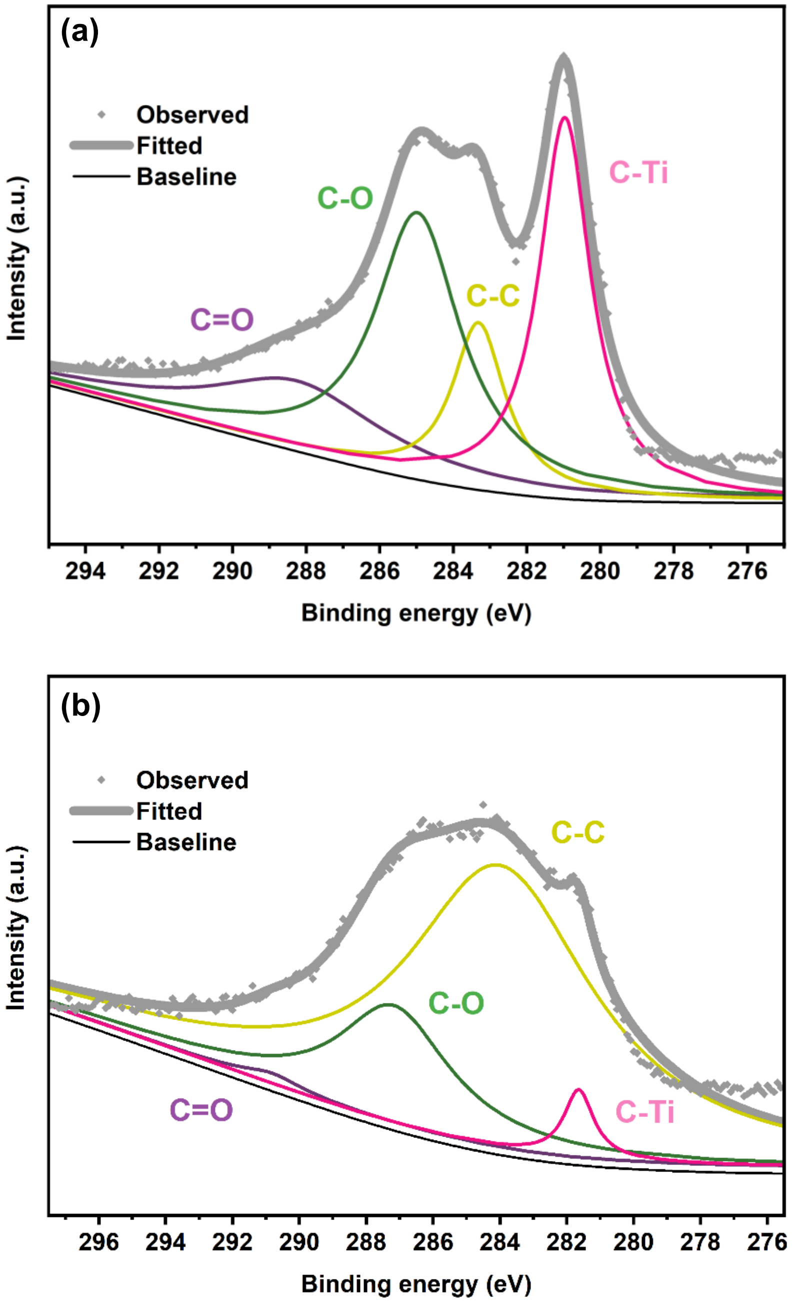

Figure S5. High-resolution C 1s peaks of (a) MX(H)/Ru-50 and (b) MX(N)/Ru-50

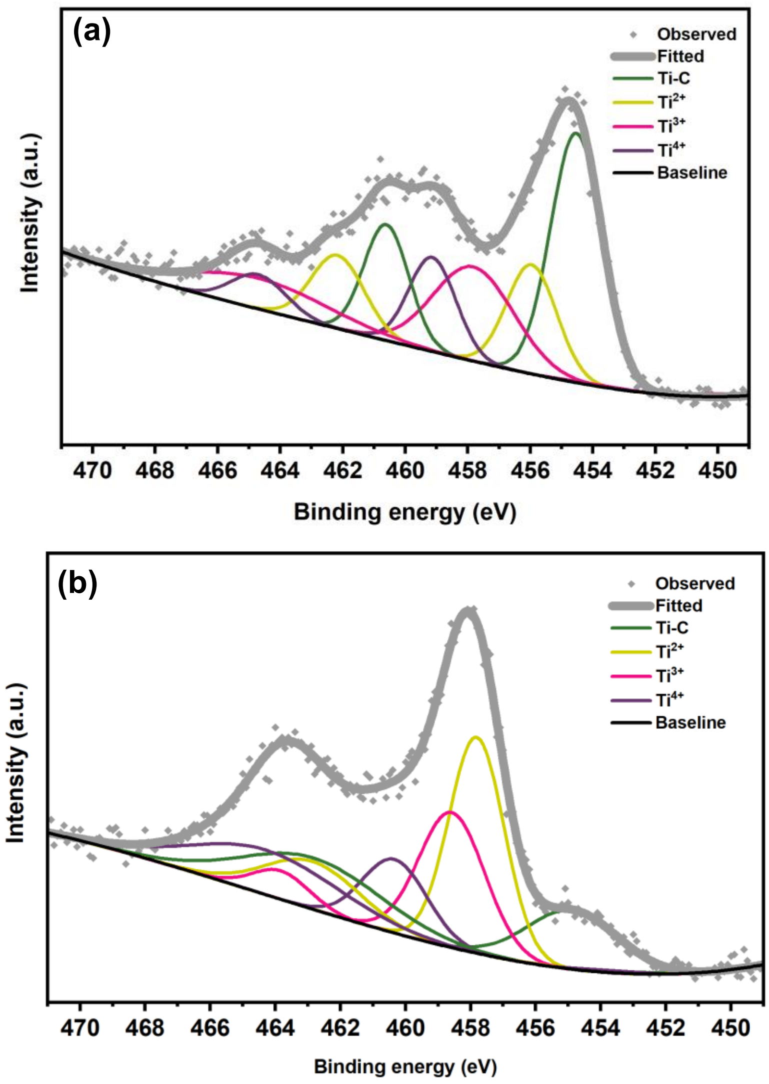

Figure S6. High-resolution Ti 2p peaks of (a) MX(H) and (b) MX(N)

The deconvoluted Ti 2p spectra of MX(H) and MX(N) show a complex overlap of multiple doublets, arising from spin-orbit splitting into Ti $2p_{3/2}$ and Ti $2p_{1/2}$ components [1]. Ti exists in multiple oxidation states due to the surface terminations that alter the local chemical environment of the surface Ti atoms. The peak at the lowest binding energy at about 455 eV corresponds to the core Ti atoms bonded directly to carbon in the MXene lattice [2–4]. The succeeding peaks show the multivalent nature (Ti^{2+}, Ti^{3+}, Ti^{4+}) of MXene which are due to Ti-F, Ti-O, and Ti-OH interactions [2–4]. The Ti-C peak in MX(N) is diminished relative to the other Ti peaks which could be attributed to abundant oxygen-rich surface terminations and the ammonium-derived oxyfluoride phase of $(NH_4)_3TiOF_5$.

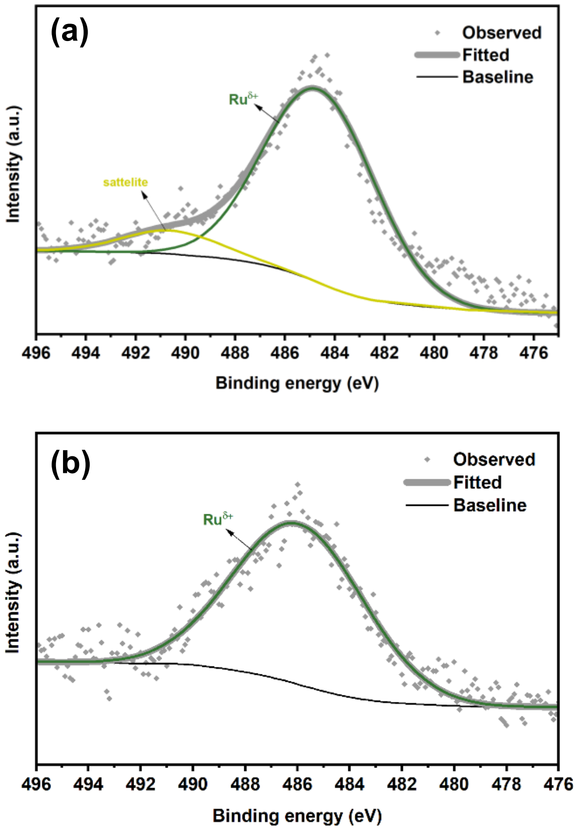

Figure S7. High-resolution Ru $3p_{1/2}$ peaks of (a) MX(H)/Ru-50 and (b) MX(N)/Ru-50

Based on prior studies and the synthesis conditions employed, the Ru species were expected to remain predominantly in their ionic state, close to +3 oxidation. In related studies, MXenes intercalated with Ru ions were examined by X-ray absorption spectroscopy. The Ru K-edge X-ray absorption near-edge structure (XANES) spectra of MXene/Ru composites were compared with Ru foil and RuO_2 references, with the composite absorption edges falling between the two standards. These results indicate that Ru in such systems exists in a partially oxidized state, $Ru^{\delta+}$ ($0 < \delta < 4$) [4,5]. The presence of a satellite peak in MX(H)/Ru-50, which appears at a higher binding energy than the primary core-level peak [6,7], suggests that it has richer electronic transitions than MX(N)/Ru-50 [7]. This feature is characteristic of a shake-up satellite, which arises when the ejection of a core electron perturbs the valence electrons. In such a process, a valence electron is promoted to an unoccupied orbital, reducing the kinetic energy of the emitted core electron relative to an electron ejected from a corresponding core orbital where shake-up has not occurred. As a result, the shake-up satellite manifests at a higher binding energy than the primary peak [7].

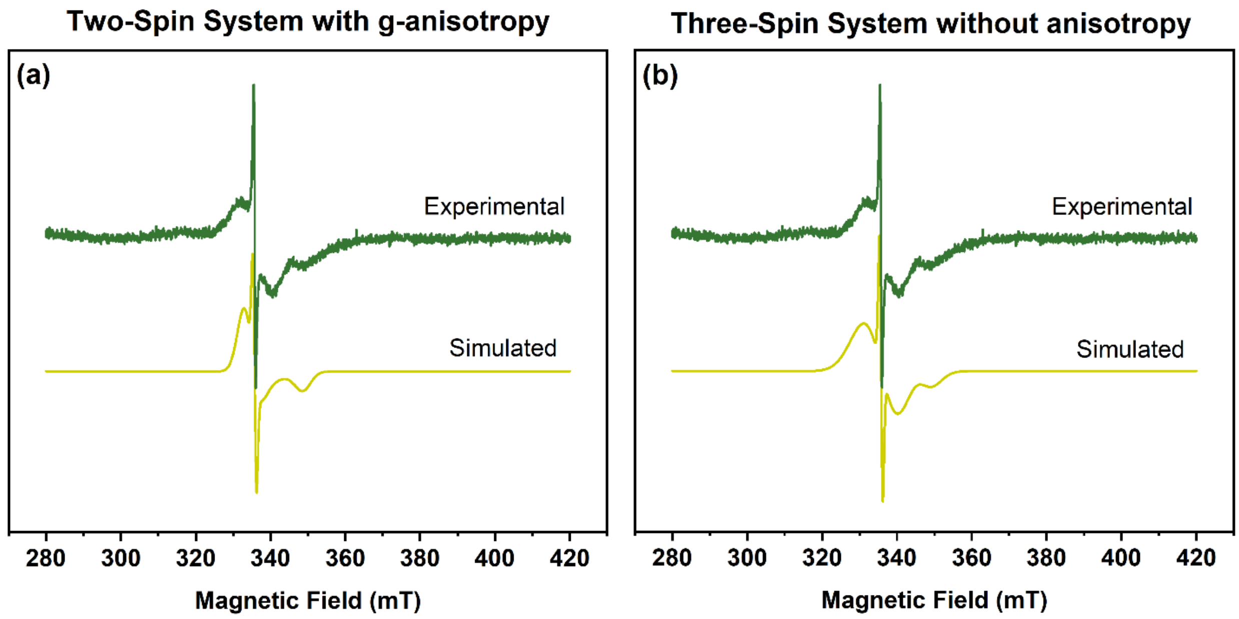

Figure S8. A comparison of the EPR spectrum for MX(H)/Ru-100 taken at 10 mW and 300 K. Simulations include: (a) A system of two-independent spins with *g*-anisotropy and (b) three independent spins with no *g*-anisotropy.

To elucidate the nature of spin centers observed in MX(H)/Ru-100, EasySpin[8] simulations were performed. The spectrum exhibited three distinguishable features: a broad central peak (~335 mT), a narrow superimposed peak at the same field, and a narrow dip near 354 mT. Initial attempts to model the spectrum using axial g-anisotropy (i.e., a single spin center producing both the broad central peak and the dip) were unsuccessful, as this configuration failed to capture the lineshape of the central peak (**Figure S8**a). However, when the dip was considered to be an independent spin center, an excellent spectral fit was achieved and easily simulated using a three-spin system without g-anisotropy (**Figure S8**b). The best-fit parameters are given in **Table S1**.

While the third spin center associated with residual Ti defects in MX(H)/Ru-100 could be confidently assigned, the remaining two spectral components present interpretive challenges. Unlike the Ti defect peak, these signals lack clear analogs in prior MXene EPR literature, leaving their precise origins ambiguous. Isolated observations from literature may offer clues. For instance, Ru nanoparticle-incorporated Ti_3C_2 synthesized using ruthenium (III) acetylacetonate and isopropyl alcohol displayed a narrow EPR signal at g = 2.0004, although the origin of this peak was not assigned.[9] Moreover, other EPR studies involving MXenes or their derivatives—particularly Ti_3C_2 exfoliated via H_2O_2 [10], MXene-derived TiO_2 nanoflowers doped with metallic

Ru [11], and MXene-derived TiO_2 composites [12]—tend to attribute observed signals to oxygen or Ti-based defects, as Ru was not contributing to the EPR signal.

Table S1. Best-fit parameters for the simulated EPR spectrum of MX(H)/Ru-100 shown in **Figure S8b**.

**Parameter**	**Spin 1 (narrow peak)**	**Spin 2 (broad central)**	**Spin 3 (Ti defect)**
g	2.0043	2.0032	1.9403
ΔH_{pp} (mT)	0.90	9.64	6.28
weight	1.11	47.78	5.10

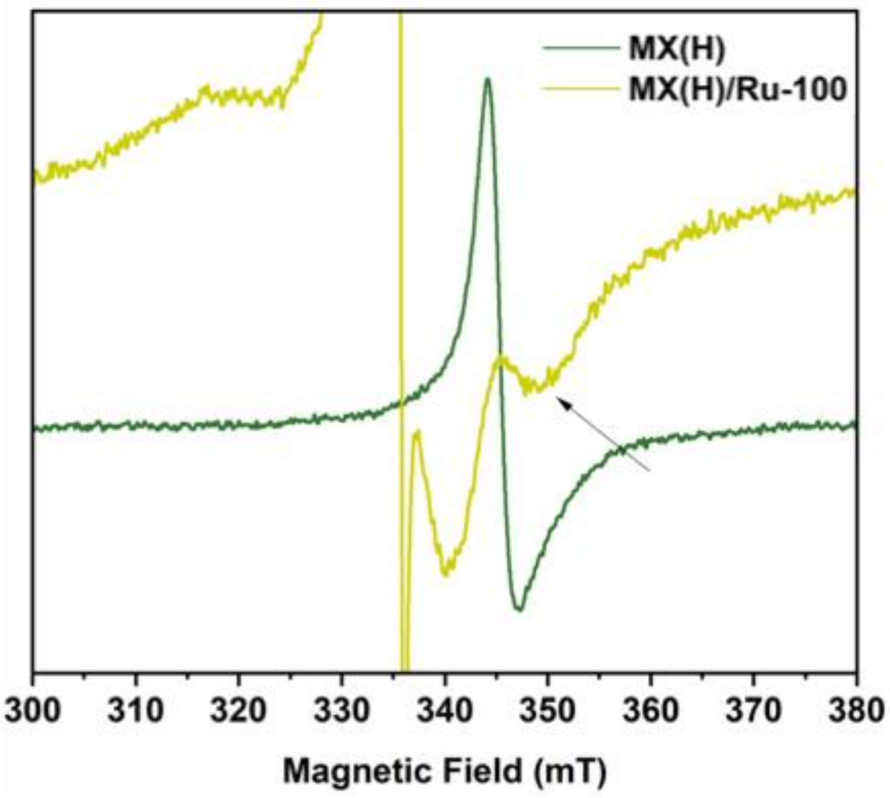

Figure S9. Zoomed in perspective of **Figure 4a** to emphasize the observed dip at around 354 mT in MX(H)/Ru-100 was very close to the peak found in its parent MX(H)

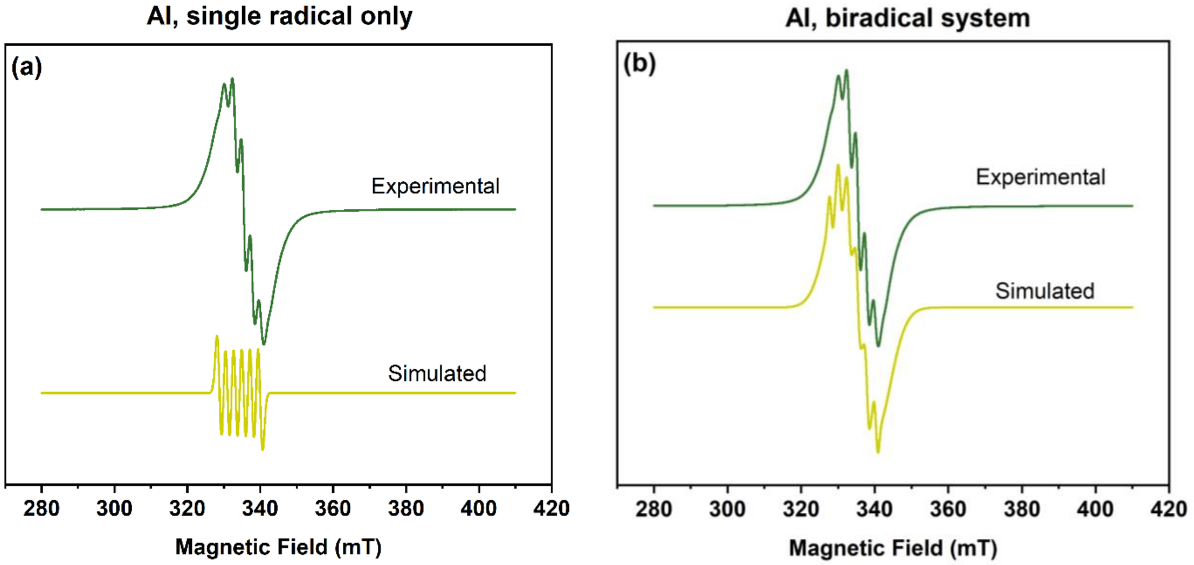

Figure S10. Attempts at simulating the X-band (9.4126 GHz) EPR spectrum of MX(N) with ^{27}Al hyperfine structure assuming (a) Interaction with a single unpaired electron and (b) Interaction with a single unpaired electron, in addition to another spin center that does not interact with ^{27}Al.

Initially, the possibility that residual ^{27}Al (*I=5/2*) contributed to the observed hyperfine structure was considered as a potential explanation for the major peaks. However, accurate reproduction of the experimental lineshapes was not achieved (**Figure S10a**) unless a second independent radical was introduced (**Figure S10b**), which did not seem very sensible. As a result, this explanation was deemed unlikely and subsequently discarded.

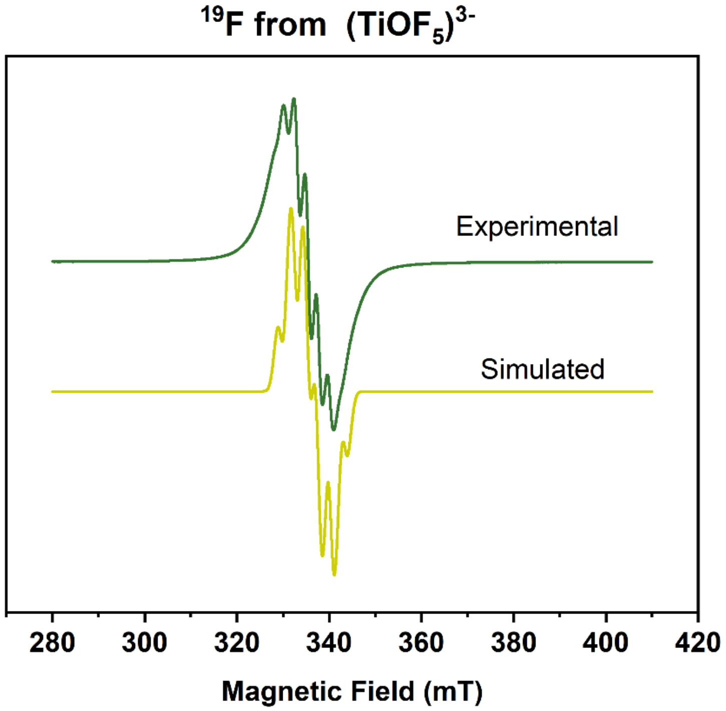

Figure S11. (a) Attempts at simulating the X-band (9.4126 GHz) EPR spectrum of MX(N) with ^{19}F hyperfine structure arising from a Ti defect for the $TiOF_5^{3-}$ anion.

A second hypothesis considered the formation of $(NH_4)_3TiOF_5$ which was indicated by the XRD patterns of MX(N). The $TiOF_5^{3-}$ anion is like a distorted octahedral geometry [13,14], and is known to undergo rotational modes and Jahn–Teller distortions [15]. Based on the local structure at room temperature of the $TiOF_5^{3-}$ anion [13], the possibility that a Ti defect in this environment might interact with neighboring ^{19}F nuclei ($I = 1/2$) was considered. While this remains speculative, it may represent a scenario analogous to Ti vacancies in HF-etched MXene, albeit occurring between layers where $(NH_4)_3TiOF_5$ has formed. However, this hypothesis did not yield a satisfactory spectral fit unless an additional independent radical was introduced and/or the hyperfine interaction between the defect and the different ^{19}F atoms were allowed to significantly vary—assumptions that lacked strong justification. Consequently, this model was not pursued further.

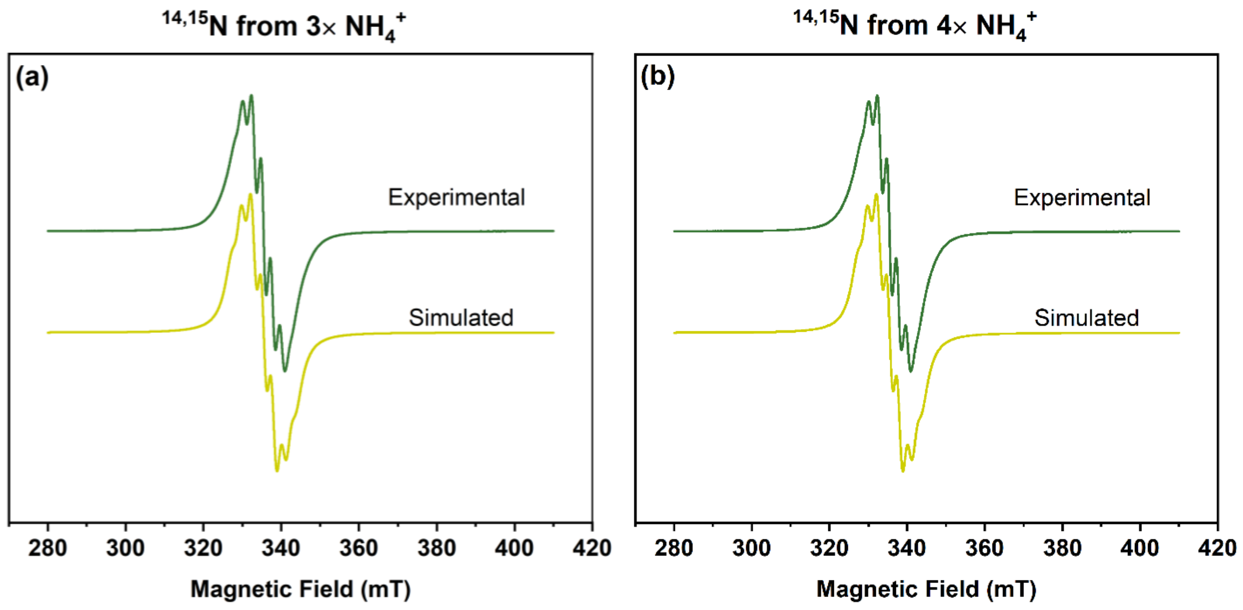

Figure S12. Attempts at simulating the X-band (9.4126 GHz) EPR spectrum of MX(N) with ^{14,15}N hyperfine structure assuming (a) three and (b) four equivalent N atoms.

Table S2. Best-fit parameters for the simulated EPR spectrum of MX(N) used in **Figure S12**

**Parameter**	**3 × ^{14,15}N**	**4 × ^{14,15}N**
g	2.004	2.004
ΔH_{pp} (mT)	2.60	2.37
N1 - A_{iso} (MHz)	70.61	66.7
N2 - A_{iso} (MHz)	70.62	66.7
N3 - A_{iso} (MHz)	70.62	66.7
N4 - A_{iso} (MHz)	--	66.6

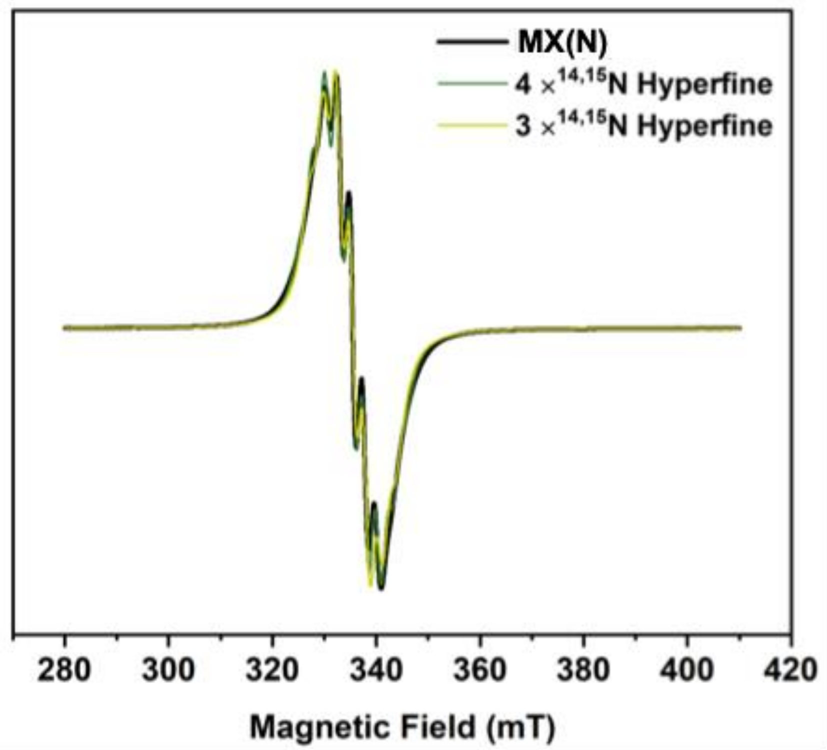

Figure S13. A direct comparison of the experimental data to simulations made with both three and four equivalent N atoms

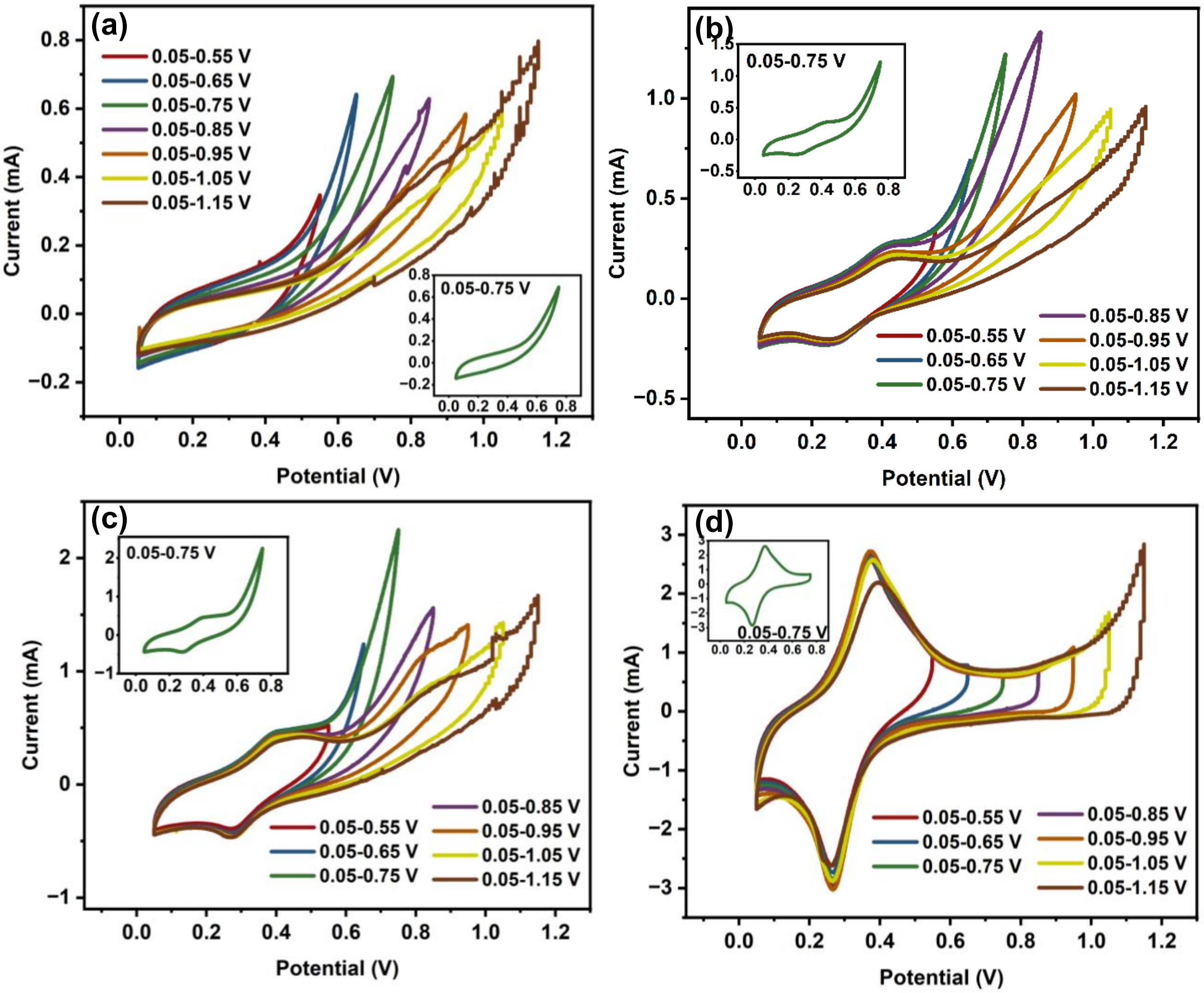

Figure S14. (a) MX(H), (b) MX(H)/Ru-5, (c) MX(H)/Ru-25, (d) MX(H)/Ru-50

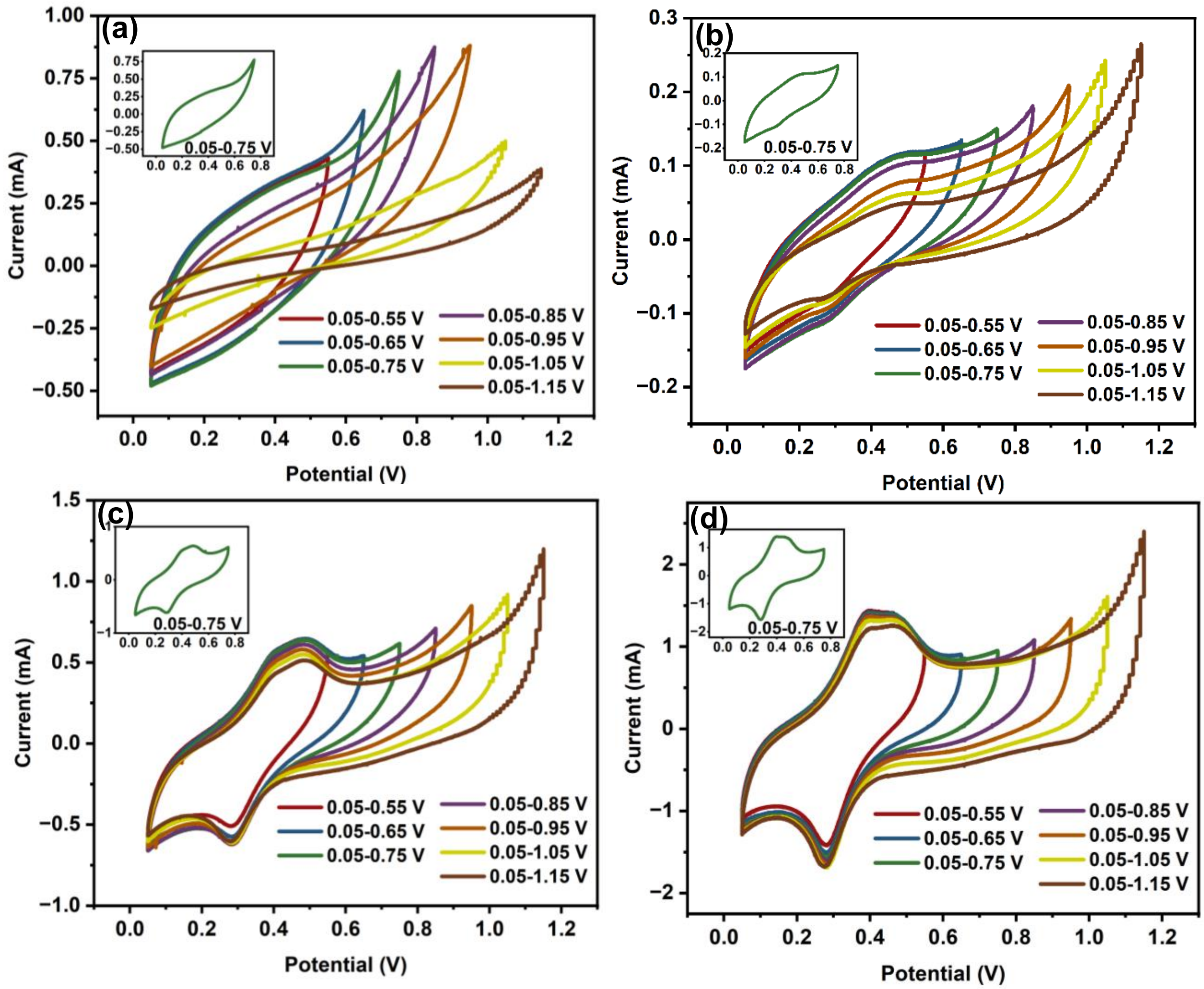

Figure S15. (a) MX(N), (b) MX(N)/Ru-5, (c) MX(N)/Ru-25, (d) MX(N)/Ru-50

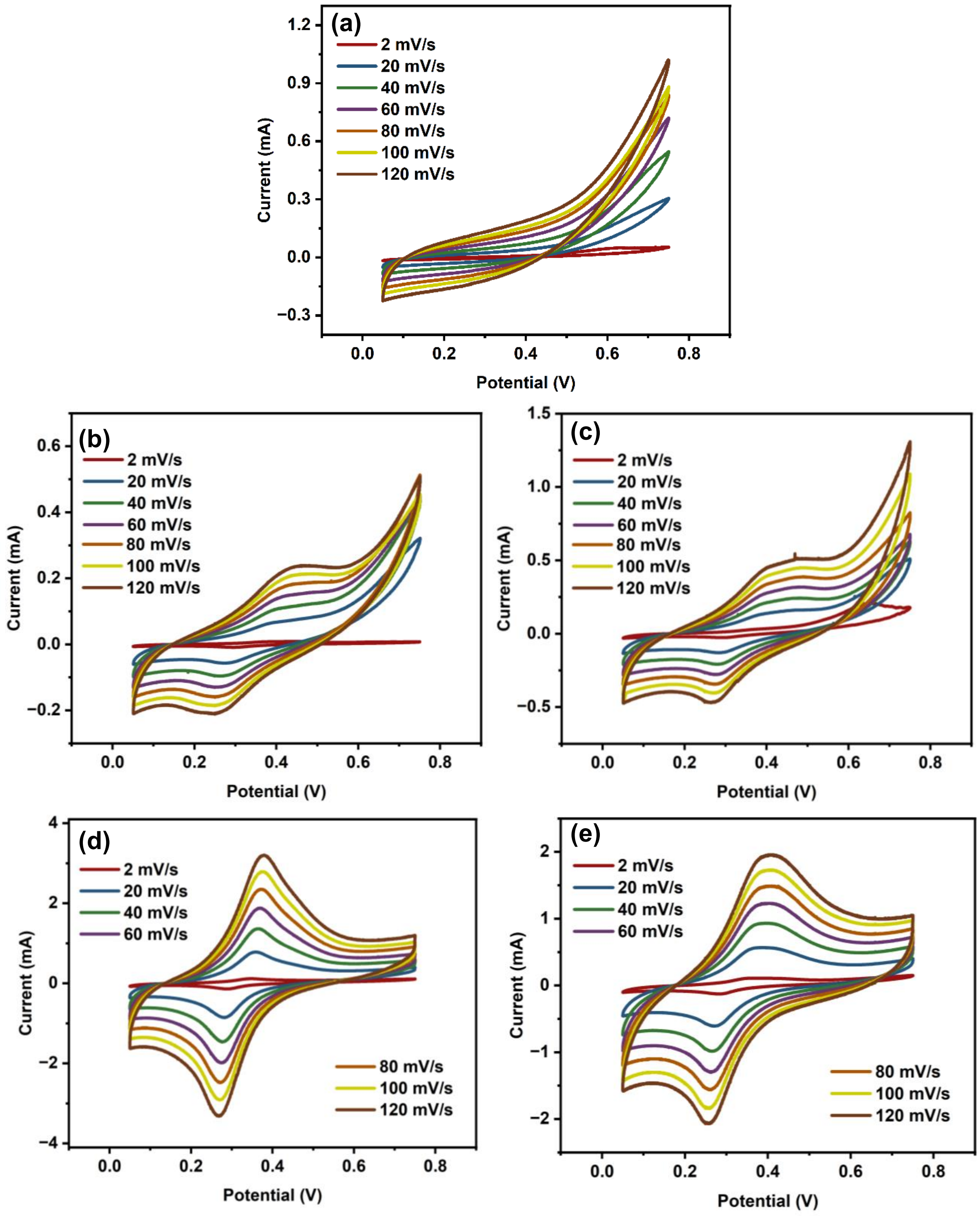

Figure S16. (a) MX(H), (b) MX(H)/Ru-5, (c) MX(H)/Ru-25, (d) MX(H)/Ru-50, (e) MX(H)/Ru-100

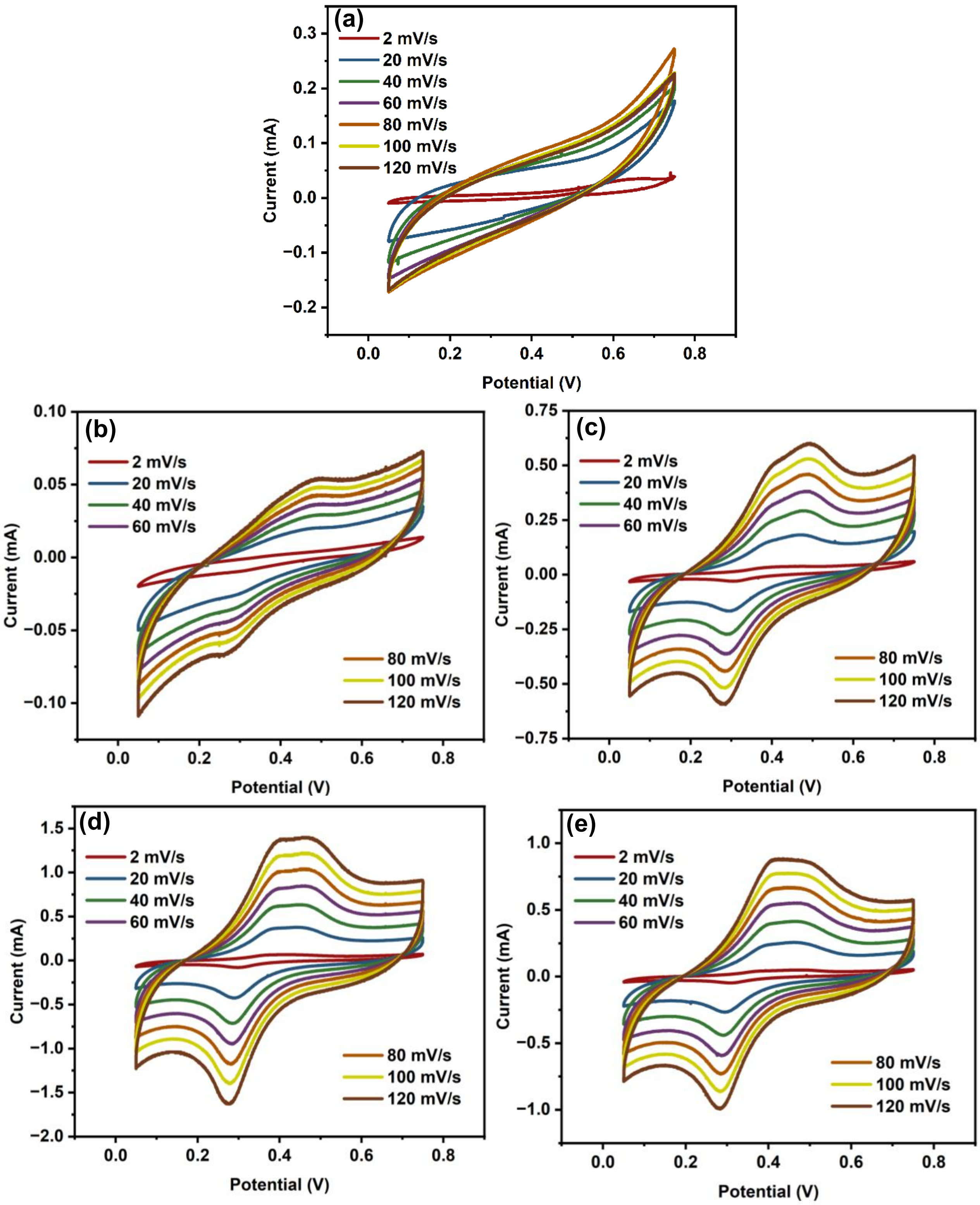

Figure S17. (a) MX(N), (b) MX(N)/Ru-5, (c) MX(N)/Ru-25, (d) MX(N)/Ru-50, (e) MX(N)/Ru-100

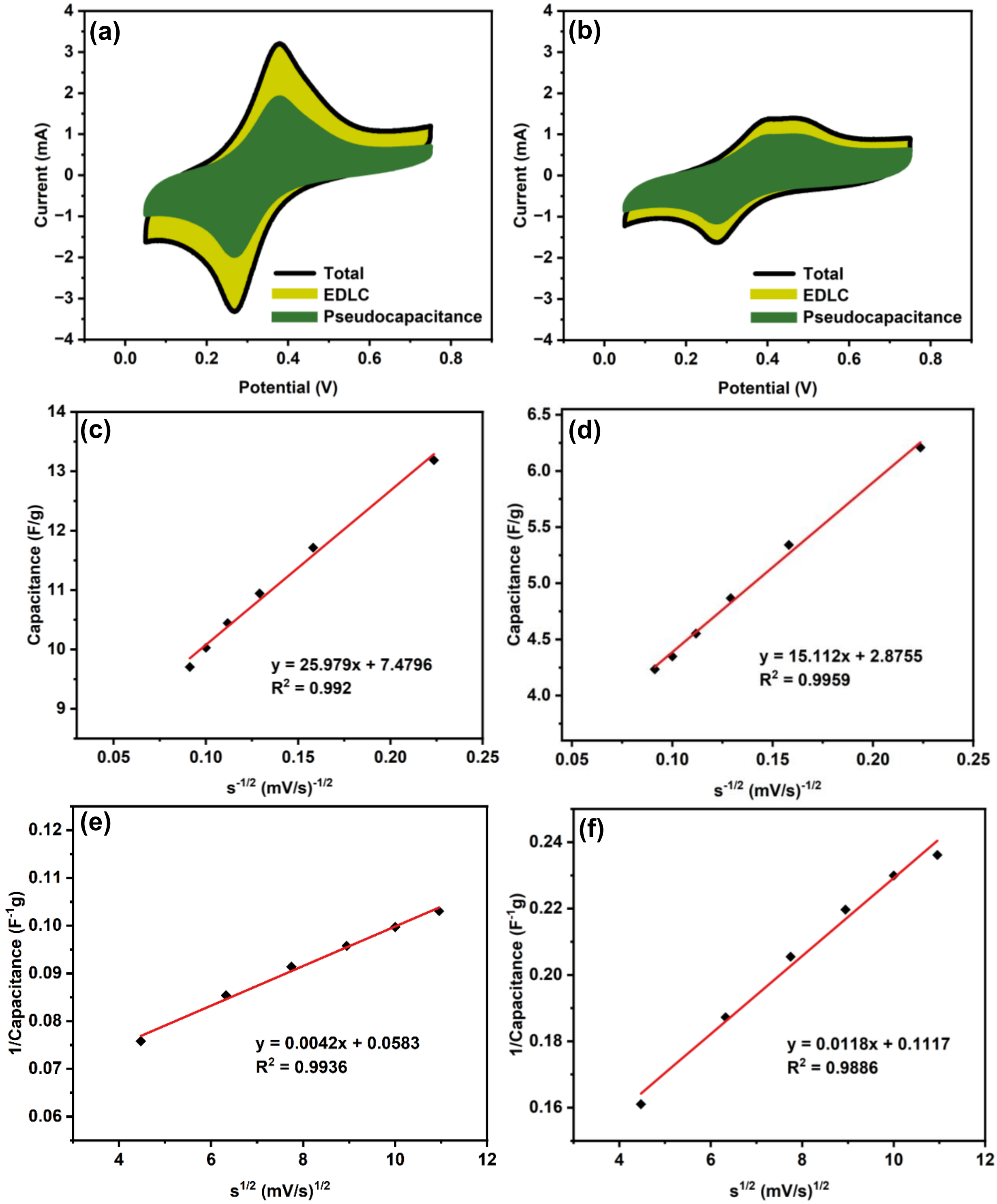

Figure S18. CV curve representations of the EDLC and pseudocapacitive contributions to the total capacitance of (a) MX(H) and (b) MX(N), as determined by Trasatti analysis; (c,d) linear fitting of $s^{-1/2}$ vs. C of MX(H) and MX(N), respectively; and (e,f) linear fitting of $s^{1/2}$ vs. $1/C$ of MX(H) and MX(N), respectively.

Table S3. Capacitance retention, initial and final capacitance values for MX(H) and MX(N) samples with varying Ru content after 8000 cycles at a fast scan rate of 200 mV/s.

**Sample**	**Capacitance Retention (%)**	**Initial Capacitance (F/g)**	**Final Capacitance (F/g)**
MX(H)	132.39	0.27	0.36
MX(H)/Ru-5	74.08	0.51	0.38
MX(H)/Ru-25	94.95	0.89	0.85
MX(H)/Ru-50	84.18	7.71	6.49
MX(H)/Ru-100	100.05	7.875	7.879
MX(N)	37.36	0.22	0.08
MX(N)/Ru-5	61.20	0.53	0.36
MX(N)/Ru-25	89.92	1.38	1.24
MX(N)/Ru-50	95.98	3.37	3.23
MX(N)/Ru-100	102.96	2.37	2.44

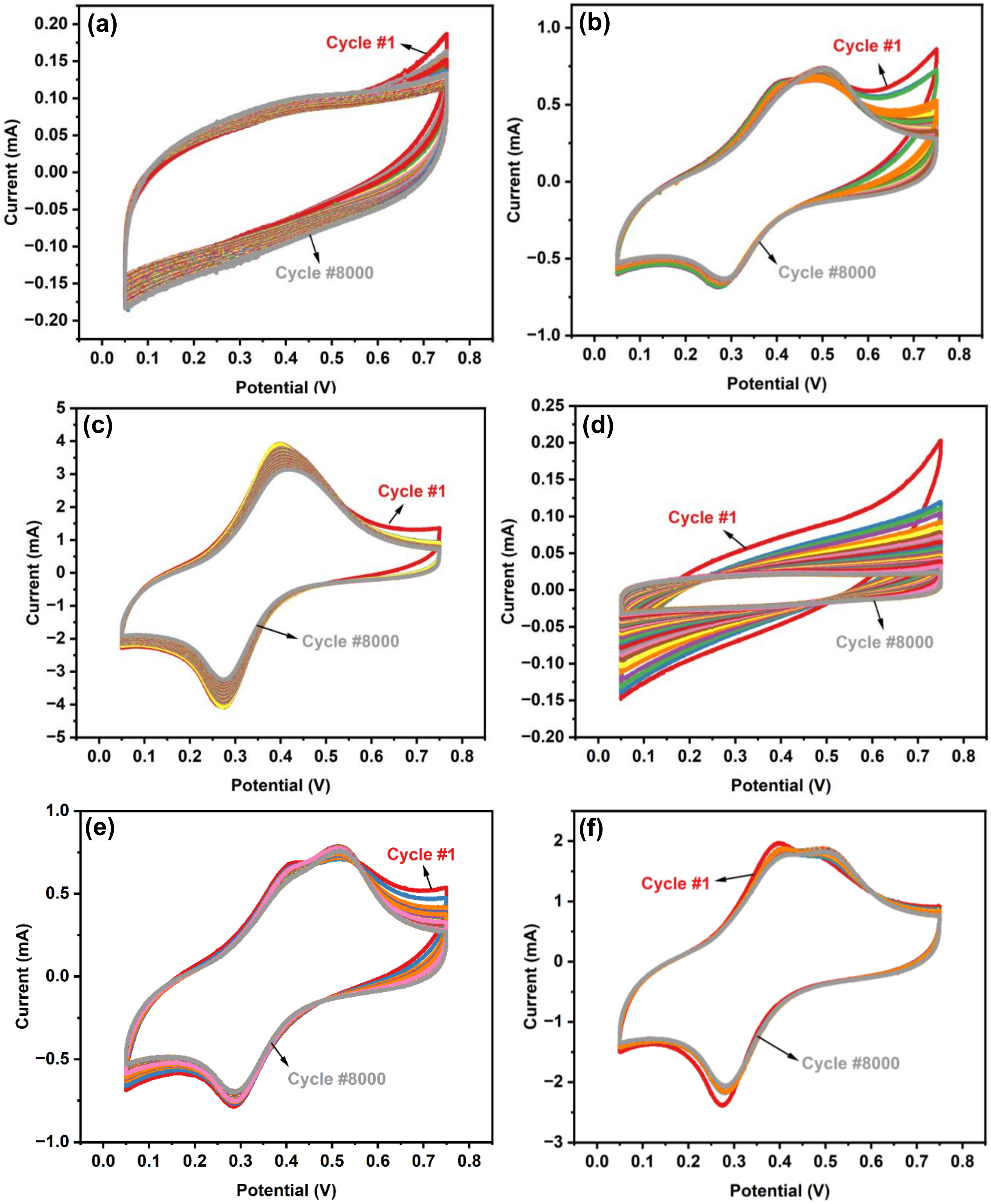

Figure S19. CV curves taken at 100-interval increments over the course of 8,000 cycles for (a) MX(H), (b) MX(H)/Ru-25, (c) MX(H)/Ru-50, (d) MX(N), (e) MX(N)/Ru-25, and (f) MX(N)/Ru-50

References

(1) Natu, V.; Benchakar, M.; Canaff, C.; Habrioux, A.; Célérier, S.; Barsoum, M. W. A Critical Analysis of the X-Ray Photoelectron Spectra of Ti3C2Tz MXenes. *Matter* **2021**, *4* (4), 1224–1251. https://doi.org/10.1016/j.matt.2021.01.015.

(2) Cao, Y.; Deng, Q.; Liu, Z.; Shen, D.; Wang, T.; Huang, Q.; Du, S.; Jiang, N.; Lin, C. Te; Yu, J. Enhanced Thermal Properties of Poly(Vinylidene Fluoride) Composites with Ultrathin Nanosheets of MXene. *RSC Adv.* **2017**, *7* (33), 20494–20501. https://doi.org/10.1039/C7RA00184C.

(3) Pahlevaninezhad, M.; Sadri, R.; Momodu, D.; Eisawi, K.; Pahlevani, M.; Naguib, M.; Roberts, E. P. L. Ammonium Bifluoride-Etched MXene Modified Electrode for TheAll−Vanadium Redox Flow Battery. *Batter. Supercaps* **2024**, *7* (4). https://doi.org/10.1002/batt.202300473.

(4) Zou, Y.; Kazemi, S. A.; Shi, G.; Liu, J.; Yang, Y.; Bedford, N. M.; Fan, K.; Xu, Y.; Fu, H.; Dong, M.; Al-Mamun, M.; Zhong, Y. L.; Yin, H.; Wang, Y.; Liu, P.; Zhao, H. Ruthenium Single-Atom Modulated Ti3C2Tx MXene for Efficient Alkaline Electrocatalytic Hydrogen Production. *EcoMat* **2023**, *5* (1). https://doi.org/10.1002/eom2.12274.

(5) Wang, X.; Ding, J.; Song, W.; Yang, X.; Zhang, T.; Huang, Z.; Wang, H.; Han, X.; Hu, W. Cation Vacancy Clusters in Ti3C2Tx MXene Induce Ultra-Strong Interaction with Noble Metal Clusters for Efficient Electrocatalytic Hydrogen Evolution. *Adv. Energy Mater.* **2023**, *13* (23), 1–11. https://doi.org/10.1002/aenm.202300148.

(6) Tiwari, J. N.; Umer, M.; Bhaskaran, G.; Umer, S.; Lee, G.; Kim, M. G.; Lee, H. K.; Kumar, K.; Vilian, A. T. E.; Huh, Y. S.; Han, Y. K. Atomic Layers of Ruthenium Oxide Coupled with Mo2TiC2Tx MXene for Exceptionally High Catalytic Activity toward Water Oxidation. *Appl. Catal. B Environ.* **2023**, *339* (June), 123139. https://doi.org/10.1016/j.apcatb.2023.123139.

(7) Brisk, M. A.; Baker, A. D. Shake-up Satellites in X-Ray Photoelectron Spectroscopy. *J. Electron Spectros. Relat. Phenomena* **1975**, *7* (3), 197–213. https://doi.org/10.1016/0368-2048(75)80061-2.

(8) Stoll, S.; Schweiger, A. EasySpin, a Comprehensive Software Package for Spectral Simulation and Analysis in EPR. *J. Magn. Reson.* **2006**, *178* (1), 42–55. https://doi.org/10.1016/j.jmr.2005.08.013.

(9) Xu, J.; Feng, Y.; Wu, P.; Tian, S.; Fang, Z.; Liu, Q.; Kong, X. Embedded Ruthenium Nanoparticles within Exfoliated Nanosheets of Ti3C2T x for Hydrogen Evolution. *ACS Appl. Nano Mater.* **2022**, *5* (10), 14241–14245.

(10) Jiang, H. J.; Underwood, T. C.; Bell, J. G.; Ranjan, S.; Sasselov, D.; Whitesides, G. M. In Situ Turning Defects of Exfoliated Ti3C2 MXene into Fenton-like Catalytic Active Sites. *Proc. Natl. Acad. Sci.* **2017**, *120*, 2017. https://doi.org/10.1073/pnas.

(11) Shi, R.; Wang, X.; Zhou, G. Electronic Metal − Support Interaction Directed Electron-Deficient Nanoparticulate Ru on Ti3C2 MXene-Derived TiO2 Nanoflowers for Robust

Benzene Semi-Hydrogenation. *Appl. Surf. Sci.* **2023**, *624* (March), 157159. https://doi.org/10.1016/j.apsusc.2023.157159.

(12) Zhou, Y.; Chai, Y.; Li, X.; Wu, Z.; Lin, J.; Han, Y.; Li, L.; Qi, H.; Gu, Y.; Kang, L.; Wang, X. Defect-Rich TiO2 in Situ Evolved from MXene for the Enhanced Oxidative Dehydrogenation of Ethane to Ethylene. *ACS Catal.* **2021**, *11* (24), 15223–15233. https://doi.org/10.1021/acscatal.1c04409.

(13) Udovenko, A. A.; Laptash, N. M. Dynamic Orientational Disorder in Crystals of Fluoroelpasolites, Structural Refinement of (NH 4) 3AlF 6, (NH 4) 3TiOF 5 and Rb 2KTiOF 5. *Acta Crystallogr. Sect. B Struct. Sci.* **2011**, *67* (6), 447–454. https://doi.org/10.1107/S0108768111044867.

(14) Udovenko, A. A.; Laptash, N. M.; Maslennikova, I. G. Orientation Disorder in Ammonium Elpasolites: Crystal Structures of (NH4)3AlF6, (NH4)3TiOF5 and (NH4)3FeF6. *J. Fluor. Chem.* **2003**, *124* (1), 5–15. https://doi.org/10.1016/S0022-1139(03)00166-0.

(15) Kavun, V. Y.; Kozlova, S. G.; Laptash, N. M.; Tkachenko, I. A.; Gabuda, S. P. Tricritical Point in Ferroelastic Ammonium Titanyl Fluoride: NMR Study. *J. Solid State Chem.* **2010**, *183* (9), 2218–2221. https://doi.org/10.1016/j.jssc.2010.07.027.
